# Effectiveness and Cost-Effectiveness of a Stratified Blended Physiotherapy Intervention Compared With Face-to-Face Physiotherapy in Patients With Nonspecific Low Back Pain: Cluster Randomized Controlled Trial

**DOI:** 10.2196/43034

**Published:** 2023-11-24

**Authors:** Tjarco Koppenaal, Johanna M van Dongen, Corelien JJ Kloek, Remco M Arensman, Cindy Veenhof, Martijn F Pisters, Raymond WJG Ostelo

**Affiliations:** 1 Research Group Empowering Healthy Behaviour Department of Health Innovations and Technology Fontys University of Applied Sciences Eindhoven Netherlands; 2 Center for Physical Therapy Research and Innovation in Primary Care Julius Health Care Centers Utrecht Netherlands; 3 Physical Therapy Research, Department of Rehabilitation, Physiotherapy Science and Sport University Medical Center Utrecht Brain Center Utrecht University Utrecht Netherlands; 4 Department of Health Sciences, Faculty of Science VU University Amsterdam Amsterdam Public Health research institute Amsterdam Netherlands; 5 Department of Health Sciences, Faculty of Science VU University Amsterdam Amsterdam Movement Sciences research institute Amsterdam Amsterdam Netherlands; 6 Research Group Innovation of Human Movement Care Research Center Healthy and Sustainable Living HU University of Applied Sciences Utrecht Netherlands; 7 Department of Epidemiology and Data Science Amsterdam University Medical Centre, Location Vrije Universiteit Amsterdam Netherlands

**Keywords:** economic evaluation, eHealth, nonspecific low back pain, physiotherapy, blended care, mobile phone

## Abstract

**Background:**

Nonspecific low back pain (LBP) is a leading contributor to disability worldwide, and its socioeconomic burden is substantial. Self-management support is an important recommendation in clinical guidelines for the physiotherapy treatment of patients with LBP and may support cost-effective management. However, providing adequate individually tailored self-management support is difficult. The integration of web-based applications into face-to-face care (ie, blended care) seems promising to optimize tailored treatment and enhance patients’ self-management and, consequently, may reduce LBP-related costs.

**Objective:**

We aimed to evaluate the long-term effectiveness and cost-effectiveness of stratified blended physiotherapy (e-Exercise LBP) compared with face-to-face physiotherapy in patients with nonspecific LBP.

**Methods:**

An economic evaluation was conducted alongside a prospective, multicenter, cluster randomized controlled trial in primary care physiotherapy. Patients with nonspecific LBP were treated with either stratified blended physiotherapy (e-Exercise LBP) (n=104) or face-to-face physiotherapy (n=104). The content of both interventions was based on the Dutch physiotherapy guidelines for nonspecific LBP. Blended physiotherapy was stratified according to the patients’ risk of developing persistent LBP using the STarT Back Screening Tool. The primary clinical outcome was physical functioning (Oswestry Disability Index version 2.1a). For the economic evaluation, quality-adjusted life years (QALYs; EQ-5D-5L) and physical functioning were the primary outcomes. Secondary clinical outcomes included fear avoidance beliefs and self-reported adherence. Costs were measured from societal and health care perspectives using self-report questionnaires. Effectiveness was estimated using linear mixed models. Seemingly unrelated regression analyses were conducted to estimate total cost and effect differences for the economic evaluation.

**Results:**

Neither clinically relevant nor statistically substantial differences were found between stratified blended physiotherapy and face-to-face physiotherapy regarding physical functioning (mean difference [MD] −1.1, 95% CI −3.9 to 1.7) and QALYs (MD 0.026, 95% CI −0.020 to 0.072) over 12 months. Regarding the secondary outcomes, fear avoidance beliefs showed a statistically significant improvement in favor of stratified blended physiotherapy (MD −4.3, 95% CI −7.3 to −1.3). Societal and health care costs were higher for stratified blended physiotherapy than for face-to-face physiotherapy, but the differences were not statistically significant (societal: €972 [US $1027], 95% CI −€1090 to €3264 [US –$1151 to $3448]; health care: €73 [US $77], 95% CI −€59 to €225 [US –$62 to $238]). Among the disaggregated cost categories, only unpaid productivity costs were significantly higher for stratified blended physiotherapy. From both perspectives, a considerable amount of money must be paid per additional QALY or 1-point improvement in physical functioning to reach a relatively low to moderate probability (ie, 0.23-0.81) of stratified blended physiotherapy being cost-effective compared with face-to-face physiotherapy.

**Conclusions:**

The stratified blended physiotherapy intervention e-Exercise LBP is neither more effective for improving physical functioning nor more cost-effective from societal or health care perspectives compared with face-to-face physiotherapy for patients with nonspecific LBP.

**Trial Registration:**

ISRCTN 94074203; https://www.isrctn.com/ISRCTN94074203

**International Registered Report Identifier (IRRID):**

RR2-10.1186/s12891-020-3174-z

## Introduction

### Background

Nonspecific low back pain (LBP) is one of the leading causes of disability and disability-adjusted life years worldwide [1-3]. Most episodes of LBP are short lasting with few consequences. However, approximately 50% of patients with LBP seen in primary care settings have a trajectory of ongoing or fluctuating low- to moderate-intensity pain, which for some develops into persistent severe LBP [4]. Recurrent episodes of LBP are common; that is, approximately 33% of patients will experience a new episode within 1 year after recovery [5]. The costs associated with health care use and productivity losses from paid work (eg, because of work absence and reduced productivity while being at work) attributed to LBP are substantial [6]. In 2017, the annual Dutch societal cost of neck pain and LBP was estimated to be €937 million (US $990 million). Health care costs, including primary care, secondary care, alternative medicine, and medication expenditures, were estimated to be approximately €878 million (US $928 million) [7]. Owing to a greater availability of improved health care technologies in combination with higher levels of spending on these technologies (higher price per unit of service), population growth, and aging, the LBP-related socioeconomic burden is expected to grow even more in the upcoming years [6,8]. This increases the need to identify cost-effective strategies for the management of LBP.

Self-management support tailored to the needs and abilities of individual patients is an important recommendation in clinical guidelines for the physiotherapy treatment of patients with LBP [9-13]. In general, this support includes advice, reassurance, and education about the nonspecific nature of LBP and the resumption of normal activities and exercise. For patients with persistent symptoms, personalized and supervised exercise therapy should be considered, possibly supported by a graded activity approach or cognitive behavioral components [14,15]. In addition to a patient-centered and stratified approach, there are indications that patients’ adherence to prescribed (home-based) exercises and recommended physical activity behavior is important for the effectiveness of care [16-19].

Web-based applications such as smartphone apps have the potential to optimize personalized face-to-face treatment and enhance patients’ self-management and adherence to prescribed management between and after face-to-face sessions [20-24]. In addition, a recent meta-analysis of randomized clinical trials concluded that smartphone and web-based self-management programs may be beneficial in improving pain and disability in patients with LBP [25]. Therefore, the integration of web-based applications into face-to-face care (ie, blended care [24]) seems to be a promising approach in the management of LBP [26].

### Objectives

To investigate whether blended care for patients with nonspecific LBP can positively influence patients’ self-management and adherence to prescribed management of LBP and consequently improve patients’ physical functioning, we developed and evaluated the stratified blended physiotherapy intervention e-Exercise LBP [27-29]. In the short term (ie, after 3 months), e-Exercise LBP was not more effective than face-to-face physiotherapy in patients with nonspecific LBP in terms of physical functioning. However, patient self-reported adherence was substantially better among patients receiving e-Exercise LBP than among those receiving face-to-face physiotherapy only [29]. Therefore, we hypothesized that, in the long term (ie, over 12 months), the stratified blended physiotherapy group patients would have improved self-management and adherence to prescribed LBP management strategies. This could lead to an improvement in physical functioning and other clinical outcomes, which in turn could result in a reduction in societal or health care costs. Therefore, this study aimed to evaluate the long-term effectiveness on physical functioning and cost-effectiveness of stratified blended care (e-Exercise LBP) compared with face-to-face physiotherapy in patients with nonspecific LBP.

## Methods

### Design Overview

An economic evaluation was conducted alongside a prospective, multicenter, cluster randomized controlled trial (RCT). Details of the design and methods of the trial have been published previously [28].

### Ethical Considerations

The Medical Research Ethics Committee of the University Medical Center Utrecht in the Netherlands approved the study protocol (18-085/D), and the study was registered at the onset of patient enrollment (ISRCTN 94074203). All participants provided written informed consent before their inclusion and took part voluntarily. The study data were handled anonymously. The trial was reported according to the CONSORT (Consolidated Standards of Reporting Trials) statement for cluster randomized trials (Multimedia Appendix 1) and the CHEERS (Consolidated Health Economic Evaluation Reporting Standards; Multimedia Appendix 2).

### Recruitment

#### Setting and Randomization

A total of 58 Dutch primary care physiotherapy practices with 122 physiotherapists were randomized at the practice level by an independent researcher according to a 1:1 allocation ratio using a computer-generated, a priori–created random sequence table. Half of the practices (29/58, 50%) were instructed to treat their patients with nonspecific LBP according to the stratified blended physiotherapy (e-Exercise LBP) protocol. The other half (29/58, 50%) treated their patients with face-to-face care following the recommendations of the guidelines for LBP of the Royal Dutch Society for Physiotherapy [30]. Physiotherapists from practices allocated to stratified blended physiotherapy received two 4-hour training sessions on e-Exercise LBP, current best evidence practice, and the study procedures. Physiotherapists from practices allocated to face-to-face physiotherapy received one 4-hour training session in current best evidence practice and the study procedures. Enrollment of patients lasted from June 2018 to December 2019, and follow-up lasted 24 months. This study evaluated the effectiveness and cost-effectiveness at the primary end point, the 12-month follow-up [28].

#### Patients

Patients were eligible if they received physiotherapy treatment for nonspecific LBP (ie, pain in the lumbosacral region, sometimes associated with pain radiating to the buttock or leg) [30], were aged ≥18 years, possessed a smartphone or tablet (iOS or Android operating system) with access to the internet, and had sufficient command of the Dutch language. Physiotherapists informed potentially eligible patients about the study and informed the research team. The research team further informed the patients about the study, verified their eligibility, and obtained written informed consent. Patients were excluded if they met any of the following criteria: a specific cause of LBP determined through medical imaging or by a medical doctor, serious comorbidities (eg, malignancy or stroke), and current pregnancy (because of the prevalence of pelvic girdle pain as a specific form of LBP).

### Intervention

#### Experimental: Stratified Blended Physiotherapy (e-Exercise LBP)

e-Exercise LBP is a stratified blended intervention in which a smartphone app is integrated into face-to-face physiotherapy treatment [27,28]. Both the content of the smartphone app and the face-to-face physiotherapy treatment are based on the recommendations of the guidelines for LBP of the Royal Dutch Society for Physiotherapy [30]. The duration and content of the stratified blended physiotherapy intervention was matched to the patients’ risk of developing persistent LBP (ie, low, medium, or high) as assessed using the Keele STarT Back Screening Tool [31]. The smartphone app contained video-supported self-management information, video-supported exercises, and a goal-oriented physical activity module. The content of both the face-to-face care and the smartphone app was tailored to the patients’ individual needs and progress by the physiotherapists (Table 1). Although physiotherapists were asked to treat patients according to the stratified blended physiotherapy protocol, they were allowed to deviate from the protocol according to their own clinical judgment. Screenshots of the smartphone app are shown in Multimedia Appendix 3.

**Table 1:** Overview of the stratified blended physiotherapy intervention (e-Exercise LBP^a^).

			Low-risk profile		Medium-risk profile		High-risk profile
**Smartphone app**							
	Duration	3 weeks		12 weeks		12 weeks	
	Information module	Knowledge-based platform with several self-management information themes (directly available)		12 weekly LBP self-management information themes, including assignments		12 weekly LBP self-management information themes, including assignments, pain education, and psychosocial risk factors	
	Exercise module	3 to 4 home-based exercises tailored to the patient’s specific functional limitations		3 to 4 home-based exercises tailored to the patient’s specific functional limitations		3 to 4 home-based exercises tailored to the patient’s specific functional limitations	
	Physical activity module	Physical activity recommendations in accordance with the LBP guidelines of the Royal Dutch Association for Physiotherapy		A 3-day baseline test to determine current level of physical activity. An 11-week, 3-time-per-week, goal-oriented training program to maintain or improve the level of physical activity. In patients avoiding physical activity because of LBP, a graded activity functionality can be activated.		A 3-day baseline test to determine current level of physical activity. An 11-week, 3-time-per-week, goal-oriented training program to maintain or improve the level of physical activity using a graded activity approach	
**Face-to-face care**							
	Sessions	2 sessions		Maximum of 8 sessions		Maximum of 12 sessions	
	Content	Reassurance, information about LBP, and instruction on self-management options and the importance of adequate physical activity behavior		Content similar to the low-risk profile content, as well as the following: the physiotherapist can consider providing evidence-based interventions (eg, passive or active joint mobilization) as recommended by the LBP guidelines of the Royal Dutch Association for Physiotherapy		Content similar to the medium-risk profile content, as well as the following: the physiotherapist addresses patients’ specific psychosocial risk factors using a cognitive behavioral approach, and pain education is provided	
**Integration of face-to-face care and smartphone app**							
	First session	Provide information about LBP and instruction on home-based exercises addressing patients’ specific functional limitations using the smartphone app		Provide information about LBP, instruction on home-based exercises addressing patients’ specific functional limitations, and instruction on 3-day baseline test using the smartphone app		Provide information about LBP, instruction on home-based exercises addressing patients’ specific functional limitations, and instruction on 3-day baseline test using the smartphone app	
	Middle sessions	N/A^b^		Evaluation of progress using the smartphone app and optimizing face-to-face care		Evaluation of progress using the smartphone app and optimizing face-to-face care	
	Final session	Evaluate progress using the smartphone app and provide recommendations to prevent recurrent episodes of LBP and maintain or improve the physical activity level		Evaluate progress using the smartphone app and provide recommendations to prevent recurrent episodes of LBP and maintain or improve the physical activity level		Evaluate progress using the smartphone app and provide recommendations to prevent recurrent episodes of LBP and maintain or improve the physical activity level	

^a^LBP: low back pain.

^b^N/A: not applicable.

#### Control: Face-to-Face Physiotherapy

Face-to-face physiotherapy was in line with the LBP guidelines of the Royal Dutch Society for Physiotherapy [30]. The guidelines distinguish 3 different patient profiles based on the clinical course of recovery (ie, normal recovery, abnormal recovery without predominant psychosocial factors, and abnormal recovery with predominant psychosocial factors) but do not use a specific tool to stratify care a priori. The content of the face-to-face physiotherapy was the same as that of the stratified blended care intervention (ie, information, exercises, and recommendations regarding physical activity). However, no recommendations or restrictions were provided regarding the number of face-to-face sessions. Physiotherapists were instructed to treat patients without using any web-based applications to ensure contrast between the 2 groups. The exact content of the therapy was left to the discretion of the physiotherapists and their clinical expertise.

### Outcome Measures

#### Overview

Primary and secondary clinical outcomes were assessed at baseline and at the 3- and 12-month follow-ups using web-based questionnaires and an accelerometer. No financial incentives were offered to complete the measurements. Reminders were sent after 7 and 14 days.

#### Primary Outcome Measures

For the effectiveness evaluation, the primary clinical outcome measure was *physical functioning*. Following the internationally accepted “Core Outcome Set” for research on patients with nonspecific LBP [32], physical functioning was assessed using the Oswestry Disability Index (ODI) version 2.1a [33]. A higher ODI score indicated increased functional disability (range 0-100).

For the economic evaluation, the primary outcomes were *physical functioning* and *health-related quality of life*. Health-related quality of life was assessed using the EQ-5D-5L [34,35]. This questionnaire comprises 5 health dimensions (mobility, self-care, usual activities, pain or discomfort, and anxiety or depression), all of which can be scored at 5 severity levels. With this, the instrument differentiates between 3125 possible health states, which were converted into utility values (range 0-1) using the Dutch tariff [36]. Quality-adjusted life years (QALYs) were calculated by multiplying the patients’ utility values by their time spent in a certain health state using linear interpolation between measurement points [37].

#### Secondary Clinical Outcome Measures

Secondary clinical outcomes included *average LBP intensity in the last week* measured using an 11-point numeric rating scale [32,38], *mean number of minutes per day spent in moderate to vigorous physical activity* objectively measured using the Activ8 accelerometer (2M Engineering) [39], *fear avoidance beliefs about physical activity and work* measured using the Fear-Avoidance Beliefs Questionnaire [40], *pain catastrophizing* measured using the Pain Catastrophizing Scale [41], *self-efficacy* measured using the General Self-Efficacy Scale [42,43], *self-management ability* assessed using the Dutch version of the short-form Patient Activation Measure [44], and *patient self-reported adherence to prescribed home exercises* measured using the Exercise Adherence Rating Scale [45]. A detailed description of the secondary clinical outcome measures can be found elsewhere [28,29].

#### Cost Outcome Measures

Costs included intervention, other health care, informal care, absenteeism, presenteeism, and unpaid productivity costs because of nonspecific LBP. Costs were assessed at 3, 6, 9, and 12 months using 3-month retrospective self-reported cost questionnaires. All costs were converted into 2020 euros using consumer price indexes [46]. Discounting of costs was not necessary because of the trial’s 12-month follow-up period.

Intervention costs were estimated based on the patients’ total number of self-reported face-to-face physiotherapy and manual therapy sessions during the first 3 months of follow-up, valued using Dutch standard costs [47]. Intervention costs also included the cost per patient for the development, hosting, and maintenance of the stratified blended physiotherapy intervention. These costs were estimated by dividing the total development, hosting, and maintenance costs (ie, €28,040 [US $29,622]) by the expected number of patients with nonspecific LBP who would be eligible for the e-Exercise LBP study during the first 5 years after implementing it broadly (ie, n=146,309) [48] and an expected implementation rate of 10%. Hence, these costs were €0.19 (US $0.20) per patient. Other health care costs included the cost of primary and secondary health care as well as medication use. Primary and secondary health care use were valued using Dutch standard costs [47]. If unavailable, prices according to professional organizations were used. Both prescribed and over-the-counter medication use were valued using unit prices derived from the Medicijnkosten.nl website [49,50]. Informal care (ie, care from family, friends, and other volunteers) was valued using a Dutch shadow price of €15.14 (US $15.99) per hour (in 2020 euros) [47]. Paid productivity losses comprised absenteeism (ie, sickness absence) and presenteeism (ie, reduced productivity while at work). Absenteeism was measured using a modified version of the Institute for Medical Technology Assessment Productivity Cost Questionnaire and valued in accordance with the “friction cost approach” (FCA) using gender-specific price weights [51,52]. The FCA assumes that costs are limited to the friction period (ie, the period needed to replace a sick worker; 85 days). Presenteeism was measured using the “Productivity and Disease Questionnaire” and valued using gender-specific price weights as well [51-53]. To assess unpaid productivity losses, patients were asked to report the number of hours that they were not able to perform volunteer work and domestic and educational activities because of their nonspecific LBP, which were valued using the same Dutch shadow price of €15.14 (US $15.99) per hour [47].

#### Baseline Measures

Baseline measures included demographic and potential confounding variables (ie, sex, age, BMI, presence of comorbidities, educational level, employment status, previous LBP surgeries, duration of LBP complaints, the presence of central sensitivity assessed using the Central Sensitization Inventory [54], and the risk of developing persistent LBP assessed using the Keele STarT Back Screening Tool [31,55]).

### Data Analysis

#### Overview

Statistical analyses were performed according to the intention-to-treat principle. Descriptive statistics were used to explore between-group baseline comparability and describe patients’ general characteristics. Using multivariate imputation by chained equations with predictive mean matching, 10 complete data sets were created (loss of efficiency of <5%) [56]. The imputation model consisted of variables that differed between groups at baseline, variables that were related to the “missingness” of data, variables associated with the outcome, and all available baseline and follow-up costs and clinical outcome measures. Each imputed data set was then analyzed separately, as specified in the following section. Pooled estimates were calculated using the rules by Rubin [57] incorporating both within-imputation variability (ie, uncertainty about the results from one imputed data set) and between-imputation variability (ie, reflecting the uncertainty because of missing information) [56]. Analysis of effectiveness and cost-effectiveness was performed using Stata (version 13.0; StataCorp LLC).

#### Analysis of Effectiveness

The effectiveness of stratified blended physiotherapy compared with face-to-face physiotherapy for the primary and secondary clinical outcomes was estimated using linear mixed models. A 2-level structure was used comprising repeated measurements (level 1) nested within patients (level 2). The necessity of using additional levels in the random-effects model to control for the clustering of patients within physiotherapy practices and individual physiotherapists was checked using log-likelihood ratios [58]. Overall mean differences (MDs) for the complete duration of follow-up, as well as MDs per time point, were estimated between stratified blended physiotherapy and face-to-face physiotherapy. Regression coefficients with 95% CIs were used to signify the differences between stratified blended physiotherapy and face-to-face physiotherapy. Analyses were adjusted for baseline values of clinical outcome measures (eg, utility score and physical functioning) and variables with a substantial difference at baseline that changed the regression coefficient for the between-group estimate by ≥10% (ie, duration of LBP complaints).

#### Analysis of Cost-Effectiveness

As indicated previously, an economic evaluation was performed from both societal and health care perspectives. When the societal perspective was applied, all costs were included. When the health care perspective was applied, only costs accruing to the formal Dutch health care sector were included.

Mean between-group cost differences were calculated for total and disaggregated costs using ordinary least squares regression analyses. Seemingly unrelated regression analyses were conducted to estimate total cost and effect differences (ie, ΔC and ΔE) while adjusting for baseline values and confounders and considering the possible correlation between costs and effects. Variables were considered confounders if they differed considerably at baseline between the groups or changed the regression coefficient by >10%. For effects, the duration of LBP complaints was a confounder. For costs, the confounders were employment status (societal perspective) and the duration of complaints (health care perspective). Incremental cost-effectiveness ratios (ICERs) were calculated by dividing the adjusted differences in total costs by the adjusted differences in effects (ie, ΔC/ΔE). Bias-corrected and accelerated bootstrapping with 5000 replications was used to estimate the uncertainty surrounding the cost differences and ICERs.

Uncertainty surrounding the ICERs was graphically illustrated by plotting bootstrapped cost-effect pairs on cost-effectiveness planes. Cost-effectiveness acceptability curves (CEACs) were constructed to indicate the probability of stratified blended physiotherapy being cost-effective in comparison with face-to-face physiotherapy at different values of willingness to pay [37]. In the Netherlands, threshold values for willingness to pay of €10,000 to €80,000 (US $10,564 to $84,513) per QALY are commonly used for societal perspective analyses [59]. For physical functioning, such threshold values are currently lacking.

#### Sensitivity Analyses

In total, 3 sensitivity analyses were conducted as part of the economic evaluation. In the first sensitivity analysis, only data from complete cases on the primary clinical and cost outcome measures were included. In the second sensitivity analysis, absenteeism costs were estimated using the human capital approach assuming that productivity losses are generated for the entire duration of absence. In the third sensitivity analysis, the analysis was performed per risk group for developing persistent LBP (low, medium, and high) separately as this proved to be an effect modifier of the between-group differences between stratified blended physiotherapy and face-to-face physiotherapy in the short term [29].

#### Sample Size

Sample size calculations were based on the recommendations of Campbell et al [60] for cluster randomized trials. To detect clinically relevant MDs between the groups at the 12-month follow-up, a difference of >6 points in physical functioning (ODI) and an SD of 14.5 were used [61-63]. In addition, repeated measures of the primary outcome during follow-up were considered, and an intraclass correlation coefficient of 0.05 was used. For the repeated measures of physical functioning, a correlation of 0.5 was estimated between baseline and follow-up measurements until the 12-month follow-up [64]. On the basis of these assumptions (power of 80%; Cronbach α=.05) and an average cluster size of 5, a total of 165 patients were needed. With an expected dropout rate of 20%, a total of 208 participating patients (n=104 per arm) were needed.

## Results

### Flow of Participants, Therapists, and Centers Through the Study

**Figure 1:**
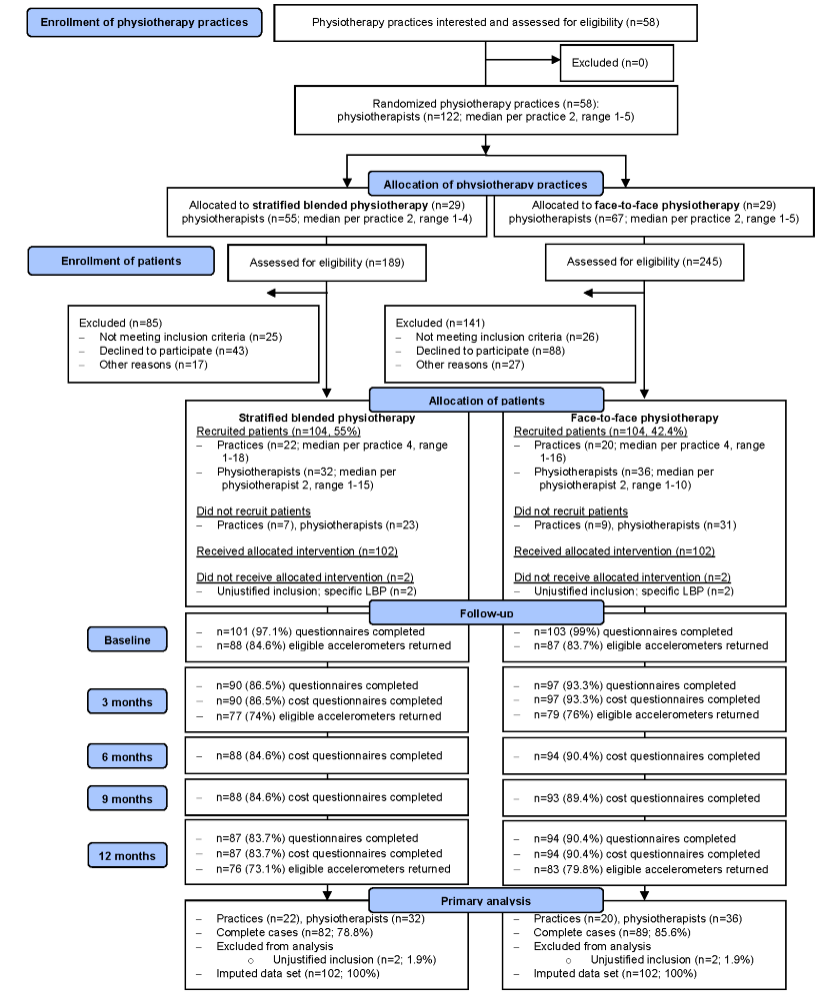
Flow diagram of the e-Exercise LBP study. LBP: low back pain.

In total, 208 eligible patients participated: 104 (50%) were allocated to the stratified blended physiotherapy group, and 104 (50%) were allocated to the face-to-face physiotherapy group (Figure 1). Complete data on all primary clinical and cost outcome measures were obtained from 82.2% (171/208) of the patients. A total of 1.9% (4/208) of the patients (2/4, 50% from the stratified blended physiotherapy group and 2/4, 50% from the face-to-face physiotherapy group) were excluded from all analyses as they were diagnosed with specific LBP immediately after inclusion and, hence, did not meet the inclusion criteria anymore. At baseline, the stratified blended physiotherapy group comprised more male individuals, more patients with a low level of education, and more patients with an LBP duration of >12 months than the face-to-face physiotherapy group. No other relevant differences in baseline characteristics were observed between the groups (Table 2).

**Table 2:** Baseline demographic and clinical characteristics of patients from the stratified blended physiotherapy group and face-to-face physiotherapy group (N=208).^a^

Characteristic		Stratified blended physiotherapy				Face-to-face physiotherapy		
		All (n=104)	Complete (n=82)	Incomplete (n=22)	All (n=104)		Complete (n=89)	Incomplete (n=15)
Sex (female), n (%)		45 (43.3)	34 (41.5)	11 (50)	57 (54.8)		51 (57.3)	6 (40)
Age (years), mean (SD)		48.1 (15.1)	50.2 (14.8)	40.4 (13.7)	47.3 (13.6)		48.1 (13.5)	42.5 (13.5)
BMI (kg/m^2^), mean (SD)		25.8 (3.8)	26.0 (3.9)	24.9 (3.2)	26.3 (5.1)		26.4 (5.2)	26.0 (4.7)
Presence of comorbidities (yes), n (%)		38 (36.5)	31 (37.8)	7 (31.8)	28 (26.9)		26 (29.2)	2 (13.3)
Employment (yes), n (%)		79 (76)	62 (75.6)	17 (77.3)	84 (80.8)		70 (78.7)	14 (93.3)
**Past LBP^b^ surgery, n (%)**								
	None	100 (96.2)	79 (96.3)	21 (95.5)	101 (97.1)		87 (97.8)	14 (93.3)
	Lumbar fusion	0 (0)	0 (0)	0 (0)	1 (1)		0 (0)	1 (6.7)
	Lumbar discectomy	4 (3.8)	3 (3.7)	1 (4.5)	2 (1.9)		2 (2.2)	0 (0)
Central sensitization (score of 0-100), mean (SD)		30.9 (13.4)	29.5 (13.3)	37.1 (12.3)	30.2 (12.2)		28.8 (11.4)	39.0 (13.6)
**Educational level, n (%)**								
	Low	22 (21.2)	15 (18.3)	7 (31.8)	13 (12.5)		12 (13.5)	1 (6.7)
	Middle	33 (31.7)	22 (26.8)	11 (50)	36 (34.6)		32 (36)	4 (26.7)
	High	49 (47.1)	45 (54.9)	4 (18.2)	55 (52.9)		45 (50.6)	10 (66.7)
**Duration of LBP complaints, n (%)**								
	0-6 weeks	37 (35.6)	30 (36.6)	7 (31.8)	49 (47.1)		39 (43.8)	10 (66.7)
	6-12 weeks	11 (10.6)	7 (8.5)	4 (18.2)	19 (18.3)		18 (20.2)	1 (6.7)
	12 weeks-12 months	9 (8.7)	7 (8.5)	2 (9.1)	9 (8.7)		7 (7.9)	2 (13.3)
	>12 months	47 (45.2)	38 (46.3)	9 (40.9)	27 (26)		25 (28.1)	2 (13.3)
**Risk of developing persistent LBP, n (%)**								
	Low	59 (56.7)	50 (61)	9 (40.9)	64 (61.5)		56 (62.9)	8 (53.3)
	Medium	35 (33.7)	26 (31.7)	9 (40.9)	37 (35.6)		31 (34.8)	6 (40)
	High	10 (9.6)	6 (7.3)	4 (18.2)	3 (2.9)		2 (2.2)	1 (6.7)
Utility score (0-1), mean (SD)		0.731 (0.188)	0.751 (0.152)	0.654 (0.278)	0.752 (0.130)		0.759 (0.131)	0.710 (0.115)
Physical functioning (score of 0-100), mean (SD)		19.4 (15.6)	18.7 (14.3)	21.7 (20.0)	20.4 (14.0)		19.9 (14.5)	23.2 (10.8)
Pain intensity (average 7-day score of 0-10), mean (SD)		5.6 (2.0)	5.5 (2.0)	6.1 (1.9)	5.4 (2.0)		5.3 (2.0)	5.7 (1.9)
Physical activity (MVPA^c^ min per day), mean (SD)		80.3 (36.8)	81.6 (37.3)	73.3 (33.9)	74.8 (40.9)		75.0 (40.7)	73.6 (44.3)
Fear avoidance beliefs (score of 0-96), mean (SD)		27.9 (16.0)	24.9 (13.9)	40.8 (18.5)	25.1 (16.2)		24.1 (15.8)	31.5 (17.8)
Pain catastrophizing (score of 0-52), mean (SD)		11.1 (9.3)	9.8 (7.9)	16.7 (12.5)	10.2 (8.7)		9.6 (8.7)	13.9 (8.3)
Self-efficacy (score of 10-40), mean (SD)		32.1 (4.4)	32.4 (4.4)	31.0 (4.3)	33.1 (3.6)		33.0 (3.8)	33.7 (2.6)
Patient activation (score of 0-100), mean (SD)		62.5 (12.4)	63.5 (11.7)	58.6 (14.2)	64.8 (12.6)		65.3 (13.1)	61.4 (8.8)

^a^Percentages may not reach 100 because of rounding.

^b^LBP: low back pain.

^c^MVPA: moderate-to-vigorous physical activity.

### Effectiveness

In the linear mixed model analyses for the primary and secondary clinical outcomes, the log-likelihood ratios of the naïve models and the models including a random intercept for both physiotherapy practice and physiotherapist were similar. Therefore, physiotherapy practice and physiotherapist were not included as a level in the linear mixed model analyses.

Both interventions were associated with improved clinical outcomes from baseline to the 12-month follow-up (within-group changes are presented in Multimedia Appendix 4). From a clinical perspective, there was neither a clinically relevant nor a statistically substantial adjusted between-group difference over 12 months in the primary outcome of physical functioning (MD −1.1, 95% CI −3.9 to 1.7). Per time point, adjusted between-group differences in physical functioning were neither clinically relevant nor statistically substantial. For most secondary clinical outcomes, there was neither a clinically relevant nor a statistically substantial adjusted between-group difference over 12 months. A statistically substantial adjusted between-group difference over 12 months and per time point was found in favor of stratified blended physiotherapy for fear avoidance beliefs (ie, overall MD −4.3, 95% CI −7.3 to −1.3; 3-month MD −3.9, 95% CI −7.5 to −0.4; 12-month MD −4.7, 95% CI −8.5 to −0.9). In addition, at the 3-month time point, a statistically substantial adjusted between-group difference was found in favor of stratified blended physiotherapy for patients’ self-reported adherence to prescribed home exercises (MD 0.8, 95% CI 0.1-1.6). Overall, differences in secondary clinical outcomes and differences per time point were not considered clinically relevant (Table 3).

**Table 3:** Adjusted overall between-group differences and adjusted between-group differences per time point for the primary and secondary clinical outcome measures.

Outcome			Stratified blended physiotherapy (n=102), mean (95% CI)		Face-to-face physiotherapy (n=102), mean (95% CI)		Adjusted between-group difference per time point^a^ (N=204), mean (95% CI)		Adjusted overall between-group difference^a^ (N=204), mean (95% CI)	
**Physical functioning (ODI^b^; 0-100)**										−1.1 (−3.9 to 1.7)
	Baseline	19.4 (16.3 to 22.4)		20.2 (17.5 to 22.9)		N/A^c^				
	3 months	9.7 (6.6 to 12.7)		9.4 (7.0 to 11.9)		−0.6 (−4.3 to 3.2)				
	12 months	8.0 (5.3 to 10.6)		8.9 (6.0 to 11.7)		−1.7 (−5.5 to 2.2)				
**Utility score (EQ-5D-5L; 0-1)**										0.026 (−0.020 to 0.072)
	Baseline	0.729 (0.692 to 0.765)		0.751 (0.725 to 0.776)		N/A				
	3 months	0.847 (0.813 to 0.880)		0.841 (0.806 to 0.876)		0.023 (−0.029 to 0.074)				
	12 months	0.851 (0.803 to 0.900)		0.840 (0.791 to 0.889)		0.029 (−0.032 to 0.090)				
**Average pain intensity in previous 7 days (NRS^d^; 0-10)**										−0.3 (−0.9 to 0.2)
	Baseline	5.7 (5.3 to 6.0)		5.4 (5.0 to 5.8)		N/A				
	3 months	3.2 (2.7 to 3.8)		3.0 (2.5 to 3.4)		0.0 (−0.7 to 0.7)				
	12 months	2.4 (1.8 to 2.9)		2.7 (2.2 to 3.2)		−0.6 (−1.3 to 0.0)				
**Physical activity (Activ8; MVPA^e^ min per day)**										2.5 (−6.9 to 11.8)
	Baseline	80.1 (71.5 to 88.7)		74.1 (65.6 to 82.7)		N/A				
	3 months	76.2 (67.0 to 85.3)		69.7 (62.1 to 77.2)		2.5 (−7.5 to 12.6)				
	12 months	76.7 (65.8 to 87.7)		70.4 (62.2 to 78.7)		2.4 (−10.6 to 15.3)				
**Fear avoidance beliefs (FABQ^f^; 0-96)**										−4.3 (−7.3 to −1.3)
	Baseline	28.1 (24.9 to 31.2)		25.4 (22.2 to 28.5)		N/A				
	3 months	23.3 (20.5 to 26.2)		25.0 (21.6 to 28.4)		−3.9 (−7.5 to −0.4)				
	12 months	21.5 (18.1 to 24.9)		24.0 (20.5 to 27.4)		−4.7 (−8.5 to −0.9)				
**Pain catastrophizing (PCS^g^; 0-52)**										−1.0 (−2.6 to 0.6)
	Baseline	11.1 (9.2 to 13.0)		10.3 (8.6 to 12.0)		N/A				
	3 months	9.1 (7.5 to 10.8)		9.3 (7.3 to 11.3)		−0.9 (−2.9 to 1.0)				
	12 months	7.9 (6.2 to 9.6)		8.2 (6.6 to 9.9)		−1.1 (−3.1 to 0.9)				
**Self-efficacy (GSE^h^ Scale; 10-40)**										0.1 (−0.8 to 1.0)
	Baseline	32.0 (31.2 to 32.9)		33.1 (32.4 to 33.8)		N/A				
	3 months	31.9 (31.0 to 32.8)		32.6 (31.9 to 33.4)		−0.0 (−1.0 to 1.0)				
	12 months	32.6 (31.7 to 33.4)		33.0 (32.2 to 33.9)		0.2 (−0.9 to 1.3)				
**Patient activation (PAM^i^ 13–Dutch; 0-100)**										0.6 (−2.4 to 3.5)
	Baseline	62.5 (60.0 to 64.9)		64.7 (62.2 to 67.2)		N/A				
	3 months	61.9 (59.5 to 64.4)		64.5 (61.9 to 67.1)		−1.5 (−4.9 to 2.0)				
	12 months	65.6 (62.5 to 68.6)		64.0 (61.2 to 66.9)		2.6 (−1.0 to 6.2)				
**Patient self-reported adherence to prescribed home exercises (EARS^j^; 0-24)^k^**										0.5 (−0.0 to 1.1)
	Baseline	—		—		N/A				
	3 months	11.9 (11.4 to 12.5)		11.1 (10.7 to 11.6)		0.8 (0.1 to 1.6)				
	12 months	12.4 (11.8 to 13.1)		12.2 (11.7 to 12.7)		0.2 (−0.5 to 1.0)				

^a^Adjusted for baseline and duration of low back pain complaints (<12 weeks vs >12 weeks).

^b^ODI: Oswestry Disability Index.

^c^N/A: not applicable.

^d^NRS: numeric rating scale.

^e^MVPA: moderate-to-vigorous physical activity.

^f^FABQ: Fear-Avoidance Beliefs Questionnaire.

^g^PCS: Pain Catastrophizing Scale.

^h^GSE: General Self-Efficacy.

^i^PAM: Patient Activation Measure.

^j^EARS: Exercise Adherence Rating Scale.

^k^Patient self-reported adherence to prescribed home exercises could only be measured after the treatment period.

### Cost-Effectiveness

#### Resource Use and Costs

Total societal (FCA) and total health care costs were higher in the stratified blended physiotherapy group (societal FCA: €5680 [US $6000], SE of the mean [SEM]=€1160 [US $1225]; health care: €512 [US $541], SEM=€64 [US $68]) than in the face-to-face physiotherapy group (societal FCA: €4851 [US $5125], SEM=€701 [US $741]; health care: €439 [US $464], SEM=€47 [US $50]). The adjusted between-group differences in total costs were not statistically substantial. Most of the disaggregated costs were highest in the stratified blended physiotherapy group. Exceptions included intervention and absenteeism costs, which were highest in the face-to-face physiotherapy group. Of all the disaggregated cost differences, only the adjusted difference in unpaid productivity costs was statistically substantial (Table 4). A detailed overview of the mean costs per participant over 12 months in the complete cases only and per risk group for developing persistent LBP is provided in Multimedia Appendix 5 for both treatment groups.

**Table 4:** Mean costs per participant in the stratified blended physiotherapy and face-to-face physiotherapy groups and the mean cost difference between groups during the 12-month follow-up period.

Cost category			Cost per participant (€), mean (SEM^a^)					Cost difference (€), mean (95% CI)		
			Stratified blended physiotherapy (n=102)		Face-to-face physiotherapy (n=102)		Crude (N=204)			Adjusted^b^ (N=204)
**Health care^c^**			512 (64)		439 (47)		73 (−60 to 220)			73 (−59 to 225)
	Intervention	210 (14)		222 (11)		−12 (−44 to 19)			−13 (−44 to 19)	
	Primary health care excluding intervention	219 (39)		148 (30)		71 (−12 to 154)			72 (−11 to 154)	
	Secondary health care	74 (33)		62 (20)		11 (−43 to 99)			12 (−43 to 102)	
	Medication	10 (3)		7 (2)		2 (−3 to 9)			2 (−4 to 9)	
Informal care			629 (162)		479 (115)		150 (−174 to 484)			143 (−185 to 475)
Absenteeism FCA^d^			773 (370)		646 (283)		127 (−607 to 929)			161 (−557 to 974)
Absenteeism HCA^e^			587 (256)		1344 (967)		−757 (−5182 to 404)			−703 (−4905 to 435)
Presenteeism			2863 (867)		2859 (536)		3 (−1446 to 1532)			131 (−1291 to 1642)
Unpaid productivity			904 (202)		427 (102)		477 (133 to 892)			464 (122 to 880)^f^
Societal FCA^g^			5680 (1160)		4851 (701)		830 (−1265 to 3124)			972 (−1090 to 3264)
Societal HCA^g^			5494 (1130)		5549 (1140)		−55 (−3500 to 2297)			108 (−3207 to 2457)

^a^SEM: SE of the mean.

^b^Adjusted for employment status (yes or no).

^c^Health care costs are the sum of the primary health care costs, secondary health care costs, medication costs, and intervention costs.

^d^FCA: friction cost approach.

^e^HCA: human capital approach.

^f^Statistically substantial difference between stratified blended physiotherapy and face-to-face physiotherapy.

^g^Societal costs are the sum of the health care costs, informal care costs, absenteeism costs, presenteeism costs, and unpaid productivity costs.

#### Effectiveness

The stratified blended physiotherapy group and face-to-face physiotherapy group gained an average of 0.834 (SEM=0.015) and 0.829 (SEM=0.016) QALYs during the 12-month follow-up, respectively. There was neither a clinically relevant nor a statistically substantial adjusted between-group difference during the 12-month follow-up period in terms of QALYs (MD 0.026, 95% CI −0.020 to 0.072; Table 3).

#### Societal Perspective

**Figure 2:**
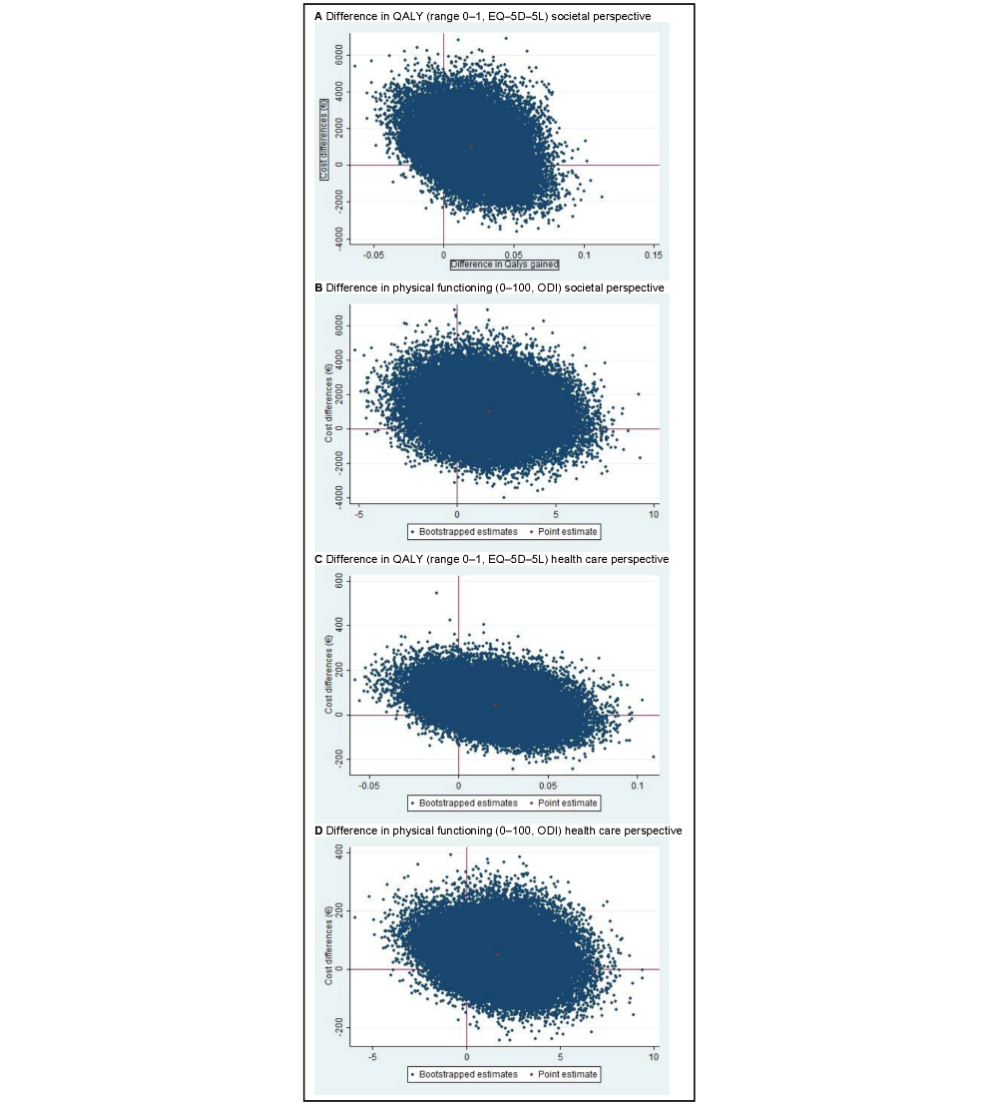
Cost-effectiveness planes from the societal and health care perspectives. ODI: Oswestry Disability Index; QALY: quality-adjusted life year.

**Figure 3:**
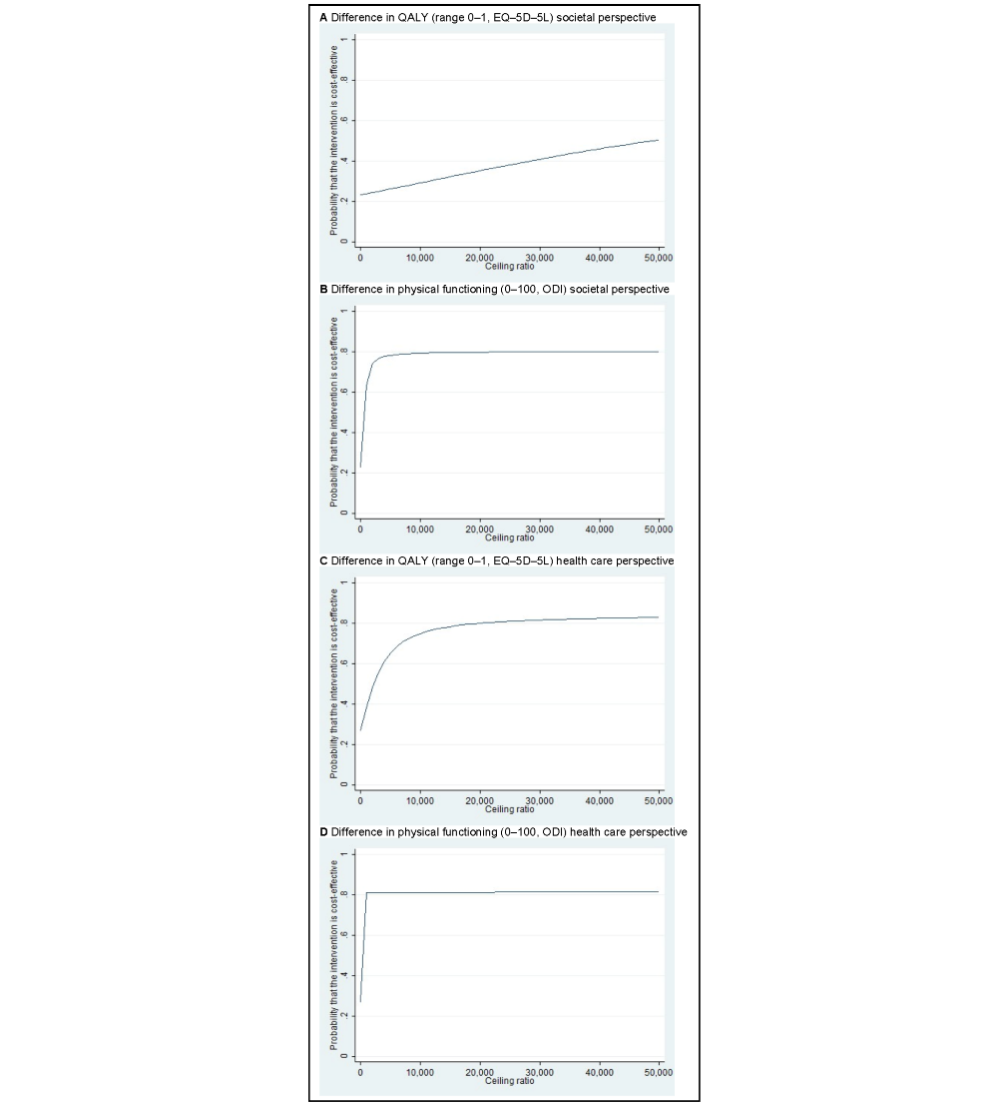
Cost-effectiveness acceptability curves from the societal and health care perspectives. ODI: Oswestry Disability Index; QALY: quality-adjusted life year.

The ICER for QALYs was 49,159, indicating that—on average—stratified blended physiotherapy was associated with an additional cost of €49,159 (US $51,932) per QALY gained compared with face-to-face physiotherapy (Table 5 and Figure 2A). The CEAC indicated that, if society is not willing to pay anything per QALY gained, the probability of stratified blended physiotherapy being cost-effective compared with face-to-face physiotherapy is 0.23 (Figure 3A). This probability increases to a maximum of 0.50 at a willingness to pay of €50,000 (US $52,821) per QALY.

For physical functioning, the ICER was −614. This indicates that stratified blended physiotherapy was—on average—associated with a societal cost of €614 (US $649) per 1-point improvement on the ODI compared with face-to-face physiotherapy (Table 5 and Figure 2B). Note that a lower ODI score indicates an improved level of physical functioning. The CEAC shows that, if decision makers are not willing to pay anything per 1-point improvement on the ODI, the probability of stratified blended physiotherapy being cost-effective compared with face-to-face physiotherapy is 0.23 (Figure 3B). This probability increases to 0.63 at a willingness to pay of €1000 (US $1056) per point improvement and to 0.79 at a willingness to pay of €10,000 (US $10,564) per point improvement.

**Table 5:** Differences in pooled mean costs and effects, incremental cost-effectiveness ratios (ICERs), and the distribution of incremental cost-effect pairs around the quadrants of the cost-effectiveness planes (N=204).

Analysis					Participants, n (%)					Outcome			ΔC^a^ (€^b^; 95% CI)			ΔE^c^ (points; 95% CI)			ICER (€ per point)			Distribution on the CE^d^ plane (%)								
					SB PT^e^		F2F PT^f^															NE^g^			SE^h^		SW^i^			NW^j^
**Main analysis (imputed data set)**																														
	Societal perspective			102 (50)		102 (50)			QALY^k^ (0-1)			994 (−1002 to 3320)			0.02 (−0.02 to 0.06)			49,159			62.2			22		1.0			14.8	
	Societal perspective			102 (50)		102 (50)			Physical functioning (ODI^l^; 0-100)			1004 (−1007 to 3306)			−1.63 (−5.43 to 2.16)			−614			60.9			19.4		3.4			16.3	
	Health care perspective			102 (50)		102 (50)			QALY (0-1)			47 (−81 to 192)			0.02 (−0.02 to 0.06)			2239			59.1			25.6		1.3			13.9	
	Health care perspective			102 (50)		102 (50)			Physical functioning (ODI; 0-100)			47 (−81 to 192)			−1.69 (−5.43 to 2.05)			−28			57.6			24.2		2.4			15.8	
**Sensitivity analysis 1—complete cases (original data set)**																														
	Societal perspective			82 (40.2)		89 (43.6)			QALY (0-1)			−385 (−1910 to 1137)			0.02 (−0.00 to 0.04)			Dominant			29.6			64.3		2.6			3.5	
	Societal perspective			82 (40.2)		89 (43.6)			Physical functioning (ODI; 0-100)			−385 (−1915 to 1133)			−2.14 (−4.80 to 0.28)			Dominant			31			64.7		1.5			2.8	
	Health care perspective			82 (40.2)		89 (43.6)			QALY (0-1)			−47 (−158 to 84)			0.02 (−0.00 to 0.43)			Dominant			21.5			73.9		2.6			2.1	
	Health care perspective			82 (40.2)		89 (43.6)			Physical functioning (ODI; 0-100)			−47 (−150 to 93)			−2.26 (−4.97 to 0.30)			Dominant			22.8			73.3		2.3			1.6	
**Sensitivity analysis 2—human capital approach (imputed data set)**																														
	Societal perspective			102 (50)		102 (50)			QALY (0-1)			136 (−3071 to 2448)			0.02 (−0.02 to 0.06)			6926			41.3			42.1		4.3			12.4	
	Societal perspective			102 (50)		102 (50)			Physical functioning (ODI; 0-100)			145 (−3126 to 2455)			−1.57 (−5.34 to 2.21)			−92			42			37.9		8			12.1	
**Sensitivity analysis 3—per risk group for developing persistent LBP^m^ (imputed data set)**																														
	**Low risk**																													
		Societal perspective	58 (28.4)			62 (30.4)		QALY (0-1)			46 (−2284 to 2492)			0.01 (−0.02 to 0.05)			3498			34.9			44.4			5.3		15.4		
		Societal perspective	58 (28.4)			62 (30.4)		Physical functioning (ODI; 0-100)			47 (−2239 to 2526)			−0.89 (−4.46 to 2.70)			−53			31.1			37.3			12.3		19.3		
		Health care perspective	58 (28.4)			62 (30.4)		QALY (0-1)			−21 (−165 to 186)			0.01 (−0.02 to 0.05)			Dominant			25.1			53			7.9		14.1		
		Health care perspective	58 (28.4)			62 (30.4)		Physical functioning (ODI; 0-100)			−21 (−162 to 193)			−0.84 (−4.38 to 2.71)			Dominant			22.5			45.1			16.2		16.3		
	**Medium risk**																													
		Societal perspective	34 (16.7)			37 (18.1)		QALY (0-1)			1124 (−2357 to 5767)			0.01 (−0.06 to 0.09)			93,372			35.9			25.7			8.6		29.8		
		Societal perspective	34 (16.7)			37 (18.1)		Physical functioning (ODI; 0-100)			1128 (−2370 to 5810)			−2.83 (−10.70 to 5.03)			−398			50.1			26.3			7.6		16.1		
		Health care perspective	34 (16.7)			37 (18.1)		QALY (0-1)			101 (−139 to 348)			0.01 (−0.06 to 0.09)			8485			43.5			17.8			4.6		34.1		
		Health care perspective	34 (16.7)			37 (18.1)		Physical functioning (ODI; 0-100)			101 (−139 to 345)			−2.83 (−10.63 to 4.98)			−36			55.6			20.4			1.7		22.3		
	**High risk**																													
		Societal perspective	10 (4.9)			3 (1.5)		QALY (0-1)			5225 (−2183 to 15,878)			0.23 (−0.25 to 0.71)			22,761			66.1			11.1			0.1		22.7		
		Societal perspective	10 (4.9)			3 (1.5)		Physical functioning (ODI; 0-100)			5182 (−2610 to 15,773)			−12.88 (−31.18 to 5.42)			−402			83.9			11.2			0.1		4.7		
		Health care perspective	10 (4.9)			3 (1.5)		QALY (0-1)			425 (130 to 896)			0.23 (−0.26 to 0.72)			1885			75.3			0.1			0		24.5		
		Health care perspective	10 (4.9)			3 (1.5)		Physical functioning (ODI; 0-100)			425 (125 to 901)			−12.81 (−30.64 to 5.02)			−33			95.1			0.2			0		4.7		

^a^C: cost.

^b^A currency exchange rate of CAD €1=US $1.05642 is applicable.

^c^E: effect.

^d^CE: cost-effectiveness.

^e^SB PT: stratified blended physiotherapy.

^f^F2F PT: face-to-face physiotherapy.

^g^Refers to the northeast quadrant of the cost-effectiveness (CE) plane, indicating that stratified blended physiotherapy (SB PT) is more effective and costlier than face-to-face physiotherapy (F2F PT).

^h^Refers to the southeast quadrant of the cost-effectiveness (CE) plane, indicating that stratified blended physiotherapy (SB PT) is more effective and less costly than face-to-face physiotherapy (F2F PT).

^i^Refers to the southwest quadrant of the cost-effectiveness (CE) plane, indicating that stratified blended physiotherapy (SB PT) is less effective and costly than face-to-face physiotherapy (F2F PT).

^j^Refers to the northwest quadrant of the cost-effectiveness (CE) plane, indicating that stratified blended physiotherapy (SB PT) is less effective and costlier than face-to-face physiotherapy (F2F PT).

^k^QALY: quality-adjusted life year.

^l^ODI: Oswestry Disability Index.

^m^LBP: low back pain.

#### Health Care Perspective

The ICER for QALYs was 2239, indicating that stratified blended physiotherapy was—on average—associated with an additional cost of €2239 (US $2365) per QALY gained compared with face-to-face physiotherapy (Table 5 and Figure 2C). The CEAC indicated that, if the health care system is not willing to pay anything per QALY gained, the probability of stratified blended physiotherapy being cost-effective compared with face-to-face physiotherapy is 0.27 (Figure 3C). This probability gradually increases to a maximum of 0.75 at a willingness to pay of €10,000 (US $10,564) per QALY.

For physical functioning, the ICER was −28. This indicates that stratified blended physiotherapy was—on average—associated with a health care cost of €28 (US $30) per 1-point improvement on the ODI compared with face-to-face physiotherapy (Table 5 and Figure 2D). Note that a lower ODI score indicates an improved level of physical functioning. The CEAC shows that, if decision makers are not willing to pay anything per 1-point improvement on the ODI, the probability of stratified blended physiotherapy being cost-effective compared with face-to-face physiotherapy is 0.27 (Figure 3D). This probability increases to 0.81 at a willingness to pay of €1000 (US $1056) per point improvement and remains the same at a higher willingness to pay.

#### Sensitivity Analysis

The direction and magnitude of the differences in costs and effects between the stratified blended physiotherapy group and the face-to-face physiotherapy group as estimated in the sensitivity analyses were not completely in line with those estimated in the main analysis. In particular, when analyzing complete cases only (sensitivity analysis 1), cost differences between the stratified blended physiotherapy group and the face-to-face physiotherapy group were found to be in favor of the stratified blended physiotherapy group, whereas when missing values were imputed (main analysis), these cost differences were in favor of the face-to-face physiotherapy group. This resulted in slightly different CEACs than those obtained in the main analysis and showed that, in the complete-case analysis, stratified blended physiotherapy was dominant over face-to-face physiotherapy. The results of sensitivity analysis 3 showed that, for both perspectives, the cost difference between the stratified blended physiotherapy group and the face-to-face physiotherapy group increased with a higher risk of developing persistent LBP; that is, for the low-, medium-, and high-risk groups, the differences in costs from the societal perspective (QALYs) were €46 (US $49), €1124 (US $1187), and €5225 (US $5520), respectively. For the low-, medium-, and high-risk groups, the differences in costs from the health care perspective (QALYs) were –€21 (US –$22), €101 (US $107), and €425 (US $449), respectively. In line with the main analysis, from the societal perspective, stratified blended physiotherapy did not seem to be cost-effective for the different risk groups for developing persistent LBP (Table 5). In addition, from the health care perspective, results for the medium- and high-risk groups were in line with the main analysis; that is, stratified blended physiotherapy did not seem to be cost-effective when compared with face-to-face physiotherapy. In the low-risk group, stratified blended physiotherapy was dominant over face-to-face physiotherapy (Table 5).

## Discussion

### Principal Findings

This study evaluated the long-term effectiveness and cost-effectiveness of the stratified blended physiotherapy intervention e-Exercise LBP in comparison with face-to-face physiotherapy in patients with nonspecific LBP. Both interventions were associated with improved clinical outcomes from baseline to the 12-month follow-up, but the study results showed neither a clinically relevant nor a statistically substantial between-group difference in the primary outcome of physical functioning and in most secondary outcomes. Over 12 months, and for each time point, only fear avoidance beliefs improved substantially more in patients who were allocated to the e-Exercise LBP group. At 3 months, patients who were allocated to the e-Exercise LBP group reported a better adherence to prescribed home exercises. However, the overall between-group difference and the differences in improvement per time point in both fear avoidance beliefs and self-reported adherence to prescribed home exercises were not considered clinically relevant. Regarding the intervention’s cost-effectiveness, from both the societal and health care perspectives, a considerable amount of money must be paid per additional QALY or 1-point improvement in physical functioning to reach a relatively low to moderate probability of e-Exercise LBP being cost-effective compared with face-to-face physiotherapy. To illustrate, e-Exercise LBP had a low probability (ie, 0.29 and 0.60) of cost-effectiveness at the upper and lower bounds, respectively, of the informal Dutch willingness-to-pay threshold for QALYs (ie, €10,000 to €80,000 [US $10,564 to $84,513] per QALY). From the health care perspective and the outcome of physical functioning, willingness-to-pay thresholds were lacking. However, we consider the maximum probability of e-Exercise LBP being cost-effective compared with face-to-face physiotherapy for both outcomes to be moderate at best (ie, <0.81). Hence, from both the societal and health care perspectives, e-Exercise LBP does not seem to be cost-effective compared with face-to-face physiotherapy among patients with nonspecific LBP. Between-group differences in costs and effects as estimated in the sensitivity analyses were not completely in line with our main analysis. In particular, the point estimates of the cost differences in the complete-case analysis were more positive than those in the main analysis and showed that e-Exercise LBP was dominant over face-to-face physiotherapy. However, the uncertainty surrounding the point estimates of the complete-case analysis was large, resulting in a relatively low probability of e-Exercise LBP being cost-effective compared with face-to-face physiotherapy, a conclusion that is similar to that of the main analysis. This was also the case for the other sensitivity analyses.

From a clinical perspective, the results of this study for the primary and secondary clinical outcomes are in line with the short-term results of the e-Exercise LBP study [29] and complement the findings of previous systematic reviews of RCTs on the added value of integrating web-based applications into the treatment of patients with LBP [23,25,26,65]. Possible explanations for the lack of short-term effectiveness (eg, the relatively large proportion of patients with a low risk of developing persistent LBP included in the analysis who have a favorable natural prognosis and the fact that blended care is not suitable for all patients) also apply to the findings of this study and have been discussed in detail previously [29]. In general, the selected contrast between the 2 studied interventions (ie, the same content delivered either face-to-face or stratified and blended) could be too small and, therefore, hamper a clear conclusion about the effectiveness of e-Exercise LBP [66,67]. Given the meaningful and comparable within-group effects in the short term for both e-Exercise LBP and face-to-face physiotherapy, an equivalence design may have been a better alternative to substantiate the possible added value of e-Exercise LBP.

Although several studies [23,26,68] have assessed the added value of integrating web-based applications into the treatment of patients with nonspecific LBP, evidence on the cost-effectiveness of such interventions is scarce. Suman et al [69] studied the cost-effectiveness of a multifaceted eHealth strategy in which face-to-face care was supported by multiple web-based components compared with a digital patient letter for patients with nonspecific LBP in primary care in the Netherlands. The reported mean societal and health care costs (€8444 [US $8920] and €1659 [US $1753]; index year 2016, respectively) and the average number of QALYs gained (0.881) during the 12-month period after receiving the intervention are comparable with our findings. The costs and effects of both treatment groups in our study are also comparable with those of other primary care physiotherapy treatments for patients with nonspecific LBP. In a review by Miyamoto et al [70] on the cost-effectiveness of exercise therapy in comparison with usual care, the number of gained QALYs during a 12-month follow-up ranged from 0.60 for physiotherapy [71] to 0.78 for exercise therapy [72]. Van de Roer et al [73] reported a mean societal cost of €4421 ([US $4670; index year 2004]) after 12 months for patients receiving face-to-face physiotherapy in line with the LBP guidelines of the Royal Dutch Society for Physiotherapy [30]. Thus, even though our stratified blended physiotherapy intervention e-Exercise LBP is not more cost-effective when compared with face-to-face physiotherapy, its costs and effects can be considered roughly the same as those of other existing primary care physiotherapy treatments for patients with nonspecific LBP. As a result, the decision regarding which intervention to administer, reimburse, or implement can be based on the preferences of the patient and the decision maker at hand. This also matches current ideas of health care policy makers regarding the integration of technology into health care [74].

A possible explanation for the lack of effectiveness and cost-effectiveness of e-Exercise LBP might be that, in contrast to our expectations, e-Exercise LBP did not result in a change in patients’ self-management behavior when compared with face-to-face physiotherapy. This is in line with our short-term results [29], and several explanations for this observation are possible. Even though integrating an app within face-to-face physiotherapy did result in substantially better self-reported adherence at the 3-month follow-up, the content of the app (ie, self-management information, integrated fortnightly reminders, and the continuing availability of the app) may have been insufficient to further support patients’ self-management behavior in the home setting. In contrast, the results of our qualitative study did reveal that patients with chronic LBP (ie, an LBP duration of >12 weeks at the start of the study) did show adequate self-management behavior when experiencing a relapse in LBP [75]. In case of a relapse, patients indicated that they first tried to gain control over their new episode of LBP before contacting a health care professional. However, patients did indicate that one of the biggest struggles was to maintain adequate health behavior in the pain-free periods between relapses in LBP. Thus, this could mean that, to facilitate long-term behavior change in patients’ management of LBP, more personalized self-management support during and after treatment is needed.

The comparison with face-to-face physiotherapy in our study might also be an important reason why we were not able to demonstrate better effectiveness and cost-effectiveness of our stratified blended physiotherapy intervention e-Exercise LBP. Despite the reasonably strong evidence that some physiotherapy interventions (compared with minimal or no intervention) for patients with nonspecific LBP are effective, the effect sizes are typically small [2,10,76,77]. As we wanted to evaluate the advantages of stratified blended physiotherapy in a pragmatic way, we decided to compare it with face-to-face physiotherapy according to the guidelines for LBP of the Royal Dutch Society for Physiotherapy [30]. As the content of both interventions was based on the guidelines, the selected between-group contrast in the delivery of treatment might have been small beforehand. In addition, given the fact that blended treatment might not be beneficial for all patients [24,78] and that this suitability was not used as an inclusion criterion, the between-group contrast might have been even smaller than expected. Consequently, a between-group difference in either effects or costs between e-Exercise LBP and face-to-face physiotherapy during the 12-month follow-up period could not be expected, either [66].

A final observation regarding our results is that presenteeism costs contributed substantially to the total societal costs (ie, 50.4% for stratified blended physiotherapy and 58.9% for face-to-face physiotherapy). This finding is in line with previous studies showing that, in many chronic conditions, presenteeism constitutes the greatest proportion of the overall associated costs [79,80]. In addition, presenteeism is a risk factor for future absenteeism and a decrement in self-rated health [81]. The distribution of total costs across the disaggregate cost categories highlights the importance of targeting presenteeism as part of future (blended) and possible cost-effective interventions for patients with nonspecific LBP.

### Strengths and Limitations

The pragmatic cluster RCT design with a follow-up period of 12 months is an important strength of this study. Such a design is acknowledged as the best setup for evaluating the effectiveness and cost-effectiveness of interventions in a real-world setting. The pragmatic approach and the involvement of 42 physiotherapy practices and 68 physiotherapists across the Netherlands improve the generalizability of the results to daily physiotherapy practice in the Netherlands [37]. A second strength is that the economic evaluation was performed from both societal and health care perspectives. In addition to the societal perspective, which is recommended in the Dutch guidelines for economic evaluation, the evaluation of the narrower health care perspective enables health care decision makers to first consider the intervention’s cost-effectiveness from their own perspective and compare this with its cost-effectiveness from the broader societal perspective. As a result, better informed decisions can be made as local policy is then considered with societal optimality in mind [82,83]. A final strength is that, in addition to QALYs as an outcome measure, physical functioning was used as an outcome measure in the economic evaluation as well. This is important as it helps understand whether the changes in QALYs are in line with the clinical effect of the studied intervention. However, note that a willingness-to-pay threshold is missing for physical functioning, making it more difficult to interpret the intervention’s cost-effectiveness in terms of this outcome.

This study also has some limitations. An important limitation is that incomplete cases had—on average—higher levels of physical functioning, lower utility scores, and higher aggregate and disaggregate costs than complete cases. This suggests that the results of the complete-case analysis are likely biased to some extent by the self-selection of patients. To address this limitation, multiple imputation, which is considered an appropriate method for imputing data that are related to observed data (ie, missing at random) and simultaneously accounts for uncertainty about the missing data by creating several imputed data sets and pooling their results, was used to handle missing data [56]. In addition, the amount of missing data in this study (ie, 14% to 21%) was relatively low compared with those in similar studies [84,85], which further improves the reliability of our multiple imputation results. As a result, greater value is attached to the results of the main analysis when compared with the complete-case analysis despite the latter being more positive. A second limitation is that stratified blended physiotherapy is still considered a “black box.” Although we provided a 2-day training for physiotherapists on the integration of the app into face-to-face physiotherapy, we have no insight into the actual fidelity of the intervention (ie, the degree to which the intervention was delivered as intended). Possibly, low fidelity contributed to the absence of effectiveness and cost-effectiveness of e-Exercise LBP compared with face-to-face physiotherapy [67]. Another limiting factor was the use of retrospective self-report questionnaires administered every 3 months to collect the cost and effect data. Self-report questionnaires are a possible source of “social desirability” or “recall bias.” However, because of the design, any recall bias or socially desirable answers are likely to have affected both groups equally, and hence, there is a small probability that the between-group differences are incorrect.

### Conclusions

This study shows that the stratified blended physiotherapy intervention e-Exercise LBP is neither more effective in terms of physical functioning nor more cost-effective from a societal or health care perspective when compared with face-to-face physiotherapy for patients with nonspecific LBP. As clinical outcomes improved in both groups from baseline to the 12-month follow-up and no statistically substantial total cost or effect differences were found between the stratified blended physiotherapy intervention e-Exercise LBP and face-to-face physiotherapy, the 2 interventions seem to be equivalent. As a result, the decision regarding which intervention should be administered or implemented can be based on the preferences of the patient and the physiotherapist or the decision maker at hand.

## Appendix

Multimedia Appendix 1 CONSORT-EHEALTH (Consolidated Standards of Reporting Trials of Electronic and Mobile Health Applications and Online Telehealth) V1.6 checklist.

Multimedia Appendix 2 CHEERS (Consolidated Health Economic Evaluation Reporting Standards) checklist.

Multimedia Appendix 3 Screenshots of the smartphone app.

Multimedia Appendix 4 Within-group changes in the stratified blended physiotherapy group and the face-to-face physiotherapy group for the primary and secondary clinical outcome measures during the 12-month follow-up period.

Multimedia Appendix 5 Mean costs per participant in the stratified blended physiotherapy group and the face-to-face physiotherapy group during the 12-month follow-up period for complete cases and per risk group for developing persistent low back pain.

## Abbreviations

CEAC: cost-effectiveness acceptability curve
CHEERS: Consolidated Health Economic Evaluation Reporting Standards
CONSORT: Consolidated Standards of Reporting Trials
FCA: friction cost approach
ICER: incremental cost-effectiveness ratio
LBP: low back pain
MD: mean difference
ODI: Oswestry Disability Index
QALY: quality-adjusted life year
RCT: randomized controlled trial
SEM: SE of the mean

## Data Availability

The data sets generated and analyzed during this study are available from the corresponding author upon reasonable request.
