# Supplementary material for: Effectiveness and Cost-Effectiveness of a Stratified Blended Physiotherapy Intervention Compared With Face-to-Face Physiotherapy in Patients With Nonspecific Low Back Pain: Cluster Randomized Controlled Trial
Source: J Med Internet Res. 2023 Nov 24;25:e43034. doi: 10.2196/43034 (PMC10709796; doi:10.2196/43034)
Supplement: Multimedia Appendix 1 [file jmir_v25i1e43034_app1.pdf]

CONSORT-EHEALTH (V 1.6.1) - Submission/Publication Form

The CONSORT-EHEALTH checklist is intended for authors of randomized trials evaluating web-based and Internet-based applications/interventions, including mobile interventions, electronic games (incl multiplayer games), social media, certain telehealth applications, and other interactive and/or networked electronic applications. Some of the items (e.g. all subitems under item 5 - description of the intervention) may also be applicable for other study designs.

The goal of the CONSORT EHEALTH checklist and guideline is to be
a) a guide for reporting for authors of RCTs,
b) to form a basis for appraisal of an ehealth trial (in terms of validity)

CONSORT-EHEALTH items/subitems are MANDATORY reporting items for studies published in the Journal of Medical Internet Research and other journals / scientific societies endorsing the checklist.

Items numbered 1., 2., 3., 4a., 4b etc are original CONSORT or CONSORT-NPT (non-pharmacologic treatment) items.
Items with Roman numerals (i., ii, iii, iv etc.) are CONSORT-EHEALTH extensions/clarifications.

As the CONSORT-EHEALTH checklist is still considered in a formative stage, we would ask that you also RATE ON A SCALE OF 1-5 how important/useful you feel each item is FOR THE PURPOSE OF THE CHECKLIST and reporting guideline (optional).

Mandatory reporting items are marked with a red *.
In the textboxes, either copy & paste the relevant sections from your manuscript into this form - please include any quotes from your manuscript in QUOTATION MARKS,
or answer directly by providing additional information not in the manuscript, or elaborating on why the item was not relevant for this study.

YOUR ANSWERS WILL BE PUBLISHED AS A SUPPLEMENTARY FILE TO YOUR PUBLICATION IN JMIR AND ARE CONSIDERED PART OF YOUR PUBLICATION (IF ACCEPTED).
Please fill in these questions diligently. Information will not be copyedited, so please use proper spelling and grammar, use correct capitalization, and avoid abbreviations.

DO NOT FORGET TO SAVE AS PDF _AND_ CLICK THE SUBMIT BUTTON SO YOUR ANSWERS ARE IN OUR DATABASE !!!

Citation Suggestion (if you append the pdf as Appendix we suggest to cite this paper in the caption):
Eysenbach G, CONSORT-EHEALTH Group
CONSORT-EHEALTH: Improving and Standardizing Evaluation Reports of Web-based and Mobile Health Interventions
J Med Internet Res 2011;13(4):e126
URL: http://www.jmir.org/2011/4/e126/
doi: 10.2196/jmir.1923

PMID: 22209829

Log in bij Google om je voortgang op te slaan. Meer informatie

*Vereist

Your name *

First Last

Tjarco Koppenaal

Primary Affiliation (short), City, Country *

University of Toronto, Toronto, Canada

Research Group Empowering Healthy Behaviou

Your e-mail address *

abc@gmail.com

t.koppenaal@fontys.nl

Title of your manuscript *

Provide the (draft) title of your manuscript.

Effectiveness and cost-effectiveness of a stratified blended physiotherapy intervention compared to face-to-face physiotherapy in patients with nonspecific low back pain: a cluster-randomized controlled trial

Name of your App/Software/Intervention *

If there is a short and a long/alternate name, write the short name first and add the long name in brackets.

e-Exercise Low Back Pain

Evaluated Version (if any)

e.g. "V1", "Release 2017-03-01", "Version 2.0.27913"

Jouw antwoord

Language(s) *

What language is the intervention/app in? If multiple languages are available, separate by comma (e.g. "English, French")

Dutch

URL of your Intervention Website or App

e.g. a direct link to the mobile app on app in appstore (itunes, Google Play), or URL of the website. If the intervention is a DVD or hardware, you can also link to an Amazon page.

https://www.e-exercise.nl/

URL of an image/screenshot (optional)

Jouw antwoord

Accessibility *

Can an enduser access the intervention presently?

- ○ access is free and open
- ◉ access only for special usergroups, not open
- ○ access is open to everyone, but requires payment/subscription/in-app purchases
- ○ app/intervention no longer accessible
- ○ Anders:

Primary Medical Indication/Disease/Condition *

e.g. "Stress", "Diabetes", or define the target group in brackets after the condition, e.g. "Autism (Parents of children with)", "Alzheimers (Informal Caregivers of)"

Low back pain

Primary Outcomes measured in trial *

comma-separated list of primary outcomes reported in the trial

he primary clinical outcome was physical func

Secondary/other outcomes

Are there any other outcomes the intervention is expected to affect?

Secondary clinical outcomes included average LBP intensity in the last week , mean number of minutes per day spent in moderate to vigorous physical activity (MVPA), fear avoidance beliefs about physical activity and work, pain catastrophizing, self-efficacy, self-management ability, patient self-reported adherence to prescribed home exercises

Recommended "Dose" *

What do the instructions for users say on how often the app should be used?

- (x) Approximately Daily
- ( ) Approximately Weekly
- ( ) Approximately Monthly
- ( ) Approximately Yearly
- ( ) "as needed"
- ( ) Anders:

Approx. Percentage of Users (starters) still using the app as recommended after 3 months *

- (selected) unknown / not evaluated
- 0-10%
- 11-20%
- 21-30%
- 31-40%
- 41-50%
- 51-60%
- 61-70%
- 71%-80%
- 81-90%
- 91-100%
- Anders:

Overall, was the app/intervention effective? *

- yes: all primary outcomes were significantly better in intervention group vs control
- partly: SOME primary outcomes were significantly better in intervention group vs control
- (selected) no statistically significant difference between control and intervention
- potentially harmful: control was significantly better than intervention in one or more outcomes
- inconclusive: more research is needed
- Anders:

Article Preparation Status/Stage *

At which stage in your article preparation are you currently (at the time you fill in this form)

- ○ not submitted yet - in early draft status
- ○ not submitted yet - in late draft status, just before submission
- ◉ submitted to a journal but not reviewed yet
- ○ submitted to a journal and after receiving initial reviewer comments
- ○ submitted to a journal and accepted, but not published yet
- ○ published
- ○ Anders:

Journal *

If you already know where you will submit this paper (or if it is already submitted), please provide the journal name (if it is not JMIR, provide the journal name under "other")

- ○ not submitted yet / unclear where I will submit this
- ◉ Journal of Medical Internet Research (JMIR)
- ○ JMIR mHealth and UHealth
- ○ JMIR Serious Games
- ○ JMIR Mental Health
- ○ JMIR Public Health
- ○ JMIR Formative Research
- ○ Other JMIR sister journal
- ○ Anders:

Is this a full powered effectiveness trial or a pilot/feasibility trial? *

- ○ Pilot/feasibility
- ◉ Fully powered

Manuscript tracking number *

If this is a JMIR submission, please provide the manuscript tracking number under "other" (The ms tracking number can be found in the submission acknowledgement email, or when you login as author in JMIR. If the paper is already published in JMIR, then the ms tracking number is the four-digit number at the end of the DOI, to be found at the bottom of each published article in JMIR)

- [x] no ms number (yet) / not (yet) submitted to / published in JMIR
- [ ] Anders:

TITLE AND ABSTRACT

1a) TITLE: Identification as a randomized trial in the title

1a) Does your paper address CONSORT item 1a? *

I.e does the title contain the phrase "Randomized Controlled Trial"? (if not, explain the reason under "other")

- [x] yes
- [ ] Anders:

1a-i) Identify the mode of delivery in the title

Identify the mode of delivery. Preferably use “web-based” and/or “mobile” and/or “electronic game” in the title. Avoid ambiguous terms like “online”, “virtual”, “interactive”. Use “Internet-based” only if Intervention includes non-web-based Internet components (e.g. email), use “computer-based” or “electronic” only if offline products are used. Use “virtual” only in the context of “virtual reality” (3-D worlds). Use “online” only in the context of “online support groups”. Complement or substitute product names with broader terms for the class of products (such as “mobile” or “smart phone” instead of “iphone”), especially if the application runs on different platforms.

	1	2	3	4	5	
subitem not at all important	○	○	◉	○	○	essential

Selectie wissen

Does your paper address subitem 1a-i? *

Copy and paste relevant sections from manuscript title (include quotes in quotation marks "like this" to indicate direct quotes from your manuscript), or elaborate on this item by providing additional information not in the ms, or briefly explain why the item is not applicable/relevant for your study

Yes, "stratified blended physiotherapy intervention"

1a-ii) Non-web-based components or important co-interventions in title

Mention non-web-based components or important co-interventions in title, if any (e.g., “with telephone support”).

	1	2	3	4	5	
subitem not at all important	○	○	●	○	○	essential

Selectie wissen

Does your paper address subitem 1a-ii?

Copy and paste relevant sections from manuscript title (include quotes in quotation marks "like this" to indicate direct quotes from your manuscript), or elaborate on this item by providing additional information not in the ms, or briefly explain why the item is not applicable/relevant for your study

Yes, "face-to-face physiotherapy"

1a-iii) Primary condition or target group in the title

Mention primary condition or target group in the title, if any (e.g., “for children with Type I Diabetes”) Example: A Web-based and Mobile Intervention with Telephone Support for Children with Type I Diabetes: Randomized Controlled Trial

	1	2	3	4	5	
subitem not at all important	○	○	●	○	○	essential

Selectie wissen

Does your paper address subitem 1a-iii? *

Copy and paste relevant sections from manuscript title (include quotes in quotation marks "like this" to indicate direct quotes from your manuscript), or elaborate on this item by providing additional information not in the ms, or briefly explain why the item is not applicable/relevant for your study

Yes, "patients with nonspecific low back pain"

1b) ABSTRACT: Structured summary of trial design, methods, results, and conclusions

NPT extension: Description of experimental treatment, comparator, care providers, centers, and blinding status.

1b-i) Key features/functionalities/components of the intervention and comparator in the METHODS section of the ABSTRACT

Mention key features/functionalities/components of the intervention and comparator in the abstract. If possible, also mention theories and principles used for designing the site. Keep in mind the needs of systematic reviewers and indexers by including important synonyms. (Note: Only report in the abstract what the main paper is reporting. If this information is missing from the main body of text, consider adding it)

	1	2	3	4	5	
subitem not at all important	○	○	○	◉	○	essential

Selectie wissen

Does your paper address subitem 1b-i? *

Copy and paste relevant sections from the manuscript abstract (include quotes in quotation marks "like this" to indicate direct quotes from your manuscript), or elaborate on this item by providing additional information not in the ms, or briefly explain why the item is not applicable/relevant for your study

Yes, "Patients with nonspecific LBP were treated with either stratified blended physiotherapy according to the e-Exercise LBP protocol (N=104) or face-to-face physiotherapy (N=104). The content of both interventions is based on the Dutch physiotherapy guidelines for nonspecific LBP. Blended physiotherapy was stratified according to the patients' risk of developing persistent LBP using the STarT Back Screening Tool."

1b-ii) Level of human involvement in the METHODS section of the ABSTRACT

Clarify the level of human involvement in the abstract, e.g., use phrases like “fully automated” vs. “therapist/nurse/care provider/physician-assisted” (mention number and expertise of providers involved, if any). (Note: Only report in the abstract what the main paper is reporting. If this information is missing from the main body of text, consider adding it)

	1	2	3	4	5	
subitem not at all important	○	○	●	○	○	essential

Selectie wissen

Does your paper address subitem 1b-ii?

Copy and paste relevant sections from the manuscript abstract (include quotes in quotation marks "like this" to indicate direct quotes from your manuscript), or elaborate on this item by providing additional information not in the ms, or briefly explain why the item is not applicable/relevant for your study

Yes, "Patients with nonspecific LBP were treated with either stratified blended physiotherapy according to the e-Exercise LBP protocol (N=104) or face-to-face physiotherapy (N=104). The content of both interventions is based on the Dutch physiotherapy guidelines for nonspecific LBP. Blended physiotherapy was stratified according to the patients’ risk of developing persistent LBP using the STarT Back Screening Tool."

1b-iii) Open vs. closed, web-based (self-assessment) vs. face-to-face assessments in the METHODS section of the ABSTRACT

Mention how participants were recruited (online vs. offline), e.g., from an open access website or from a clinic or a closed online user group (closed usergroup trial), and clarify if this was a purely web-based trial, or there were face-to-face components (as part of the intervention or for assessment). Clearly say if outcomes were self-assessed through questionnaires (as common in web-based trials). Note: In traditional offline trials, an open trial (open-label trial) is a type of clinical trial in which both the researchers and participants know which treatment is being administered. To avoid confusion, use “blinded” or “unblinded” to indicated the level of blinding instead of “open”, as “open” in web-based trials usually refers to “open access” (i.e. participants can self-enrol). (Note: Only report in the abstract what the main paper is reporting. If this information is missing from the main body of text, consider adding it)

	1	2	3	4	5	
subitem not at all important	○	○	◉	○	○	essential

Selectie wissen

Does your paper address subitem 1b-iii?

Copy and paste relevant sections from the manuscript abstract (include quotes in quotation marks "like this" to indicate direct quotes from your manuscript), or elaborate on this item by providing additional information not in the ms, or briefly explain why the item is not applicable/relevant for your study

Yes, "Patients with nonspecific LBP were treated with either stratified blended physiotherapy according to the e-Exercise LBP protocol (N=104) or face-to-face physiotherapy (N=104). The content of both interventions is based on the Dutch physiotherapy guidelines for nonspecific LBP. Blended physiotherapy was stratified according to the patients’ risk of developing persistent LBP using the STarT Back Screening Tool."

1b-iv) RESULTS section in abstract must contain use data

Report number of participants enrolled/assessed in each group, the use/uptake of the intervention (e.g., attrition/adherence metrics, use over time, number of logins etc.), in addition to primary/secondary outcomes. (Note: Only report in the abstract what the main paper is reporting. If this information is missing from the main body of text, consider adding it)

	1	2	3	4	5	
subitem not at all important	○	○	◉	○	○	essential

Selectie wissen

Does your paper address subitem 1b-iv?

Copy and paste relevant sections from the manuscript abstract (include quotes in quotation marks "like this" to indicate direct quotes from your manuscript), or elaborate on this item by providing additional information not in the ms, or briefly explain why the item is not applicable/relevant for your study

Yes, "Patients with nonspecific LBP were treated with either stratified blended physiotherapy according to the e-Exercise LBP protocol (N=104) or face-to-face physiotherapy (N=104)." AND "Neither clinically relevant, nor statistically significant, differences were found between stratified blended physiotherapy and face-to-face physiotherapy in terms of physical functioning (MD: -1.1; 95% CI -3.9 to 1.7) and QALYs (MD: 0.026; 95% CI -0.020 to 0.072) over 12 months. As for the secondary outcomes, fear avoidance beliefs showed a statistically significant improvement in favor of stratified blended physiotherapy. Societal and healthcare costs were higher for stratified blended physiotherapy than for face-to-face physiotherapy, but differences were not statistically significant (societal: €972, 95% CI -1090 to 3264; healthcare: €73, 95% CI -59 to 225). Of the disaggregated cost categories, only unpaid productivity costs were statistically significantly higher for stratified blended physiotherapy. From both perspectives, a considerable amount of money must be paid per additional QALY or 1-point improvement in physical functioning to reach a relatively low to moderate probability (i.e., 0.23 to 0.81) of stratified blended physiotherapy being cost-effective compared to face-to-face physiotherapy, respectively."

1b-v) CONCLUSIONS/DISCUSSION in abstract for negative trials

Conclusions/Discussions in abstract for negative trials: Discuss the primary outcome - if the trial is negative (primary outcome not changed), and the intervention was not used, discuss whether negative results are attributable to lack of uptake and discuss reasons. (Note: Only report in the abstract what the main paper is reporting. If this information is missing from the main body of text, consider adding it)

	1	2	3	4	5	
subitem not at all important	○	○	◉	○	○	essential

Selectie wissen

Does your paper address subitem 1b-v?

Copy and paste relevant sections from the manuscript abstract (include quotes in quotation marks "like this" to indicate direct quotes from your manuscript), or elaborate on this item by providing additional information not in the ms, or briefly explain why the item is not applicable/relevant for your study

Yes, "The stratified blended physiotherapy intervention e-Exercise LBP is neither more effective for improving physical functioning, nor more cost-effective from societal or healthcare perspectives when compared to face-to-face physiotherapy for patients with nonspecific LBP."

INTRODUCTION

2a) In INTRODUCTION: Scientific background and explanation of rationale

2a-i) Problem and the type of system/solution

Describe the problem and the type of system/solution that is object of the study: intended as stand-alone intervention vs. incorporated in broader health care program? Intended for a particular patient population? Goals of the intervention, e.g., being more cost-effective to other interventions, replace or complement other solutions? (Note: Details about the intervention are provided in “Methods” under 5)

	1	2	3	4	5	
subitem not at all important	○	○	◉	○	○	essential

Selectie wissen

Does your paper address subitem 2a-i? *

Copy and paste relevant sections from the manuscript (include quotes in quotation marks "like this" to indicate direct quotes from your manuscript), or elaborate on this item by providing additional information not in the ms, or briefly explain why the item is not applicable/relevant for your study

Yes, "approximately 50% of patients with LBP seen in primary care settings have a trajectory of ongoing or fluctuating low- to moderate-intensity pain, which for some develops into persistent severe LBP4. Recurrent episodes of LBP are common. That is, approximately 33% of patients will experience a new episode within one year after recovery5. The costs associated with healthcare use and productivity losses from paid work, e.g., due to work absence and reduced productivity while being at work, attributed to LBP are enormous6. In 2017, the annual Dutch societal cost of neck pain and LBP was estimated to be 937 million Euros. Healthcare costs, including primary care, secondary care, alternative medicine, and medication expenditures, were estimated to be approximately 878 million Euros7. Due to a greater availability of improved healthcare technologies in combination with higher levels of spending on these technologies (higher price per unit of service), population growth and aging, the LBP-related socioeconomic burden is expected to grow even more in the upcoming years6,8. This increases the need to identify cost-effective strategies for the management of LBP."

2a-ii) Scientific background, rationale: What is known about the (type of) system

Scientific background, rationale: What is known about the (type of) system that is the object of the study (be sure to discuss the use of similar systems for other conditions/diagnoses, if appropiate), motivation for the study, i.e. what are the reasons for and what is the context for this specific study, from which stakeholder viewpoint is the study performed, potential impact of findings [2]. Briefly justify the choice of the comparator.

	1	2	3	4	5	
subitem not at all important	○	○	◉	○	○	essential

Selectie wissen

Does your paper address subitem 2a-ii? *

Copy and paste relevant sections from the manuscript (include quotes in quotation marks "like this" to indicate direct quotes from your manuscript), or elaborate on this item by providing additional information not in the ms, or briefly explain why the item is not applicable/relevant for your study

Yes, "Online applications, such as smartphone apps, have the possibility of optimizing personalized face-to-face treatment and enhancing patients' self-management and adherence to prescribed management between and after face-to-face sessions20–24. Additionally, a recent meta-analysis of randomized clinical trials concluded that smartphone and web-based self-management programs may be beneficial in improving pain and disability in patients with LBP25. Therefore, the integration of online applications into face-to-face care, i.e., blended care24, seems to be a promising approach in the management of LBP26.

To investigate whether blended care for patients with nonspecific LBP can positively influence patients' self-management and adherence to prescribed management of LBP and consequently improve patients' physical functioning, we developed and evaluated the stratified blended physiotherapy intervention e-Exercise LBP27–29. In the short-term, i.e., after 3 months, e-Exercise LBP was not more effective than face-to-face physiotherapy in patients with nonspecific LBP in terms of physical functioning. However, patient self-reported adherence was significantly better among patients receiving e-Exercise LBP than among those receiving face-to-face physiotherapy only29."

2b) In INTRODUCTION: Specific objectives or hypotheses

Does your paper address CONSORT subitem 2b? *

Copy and paste relevant sections from the manuscript (include quotes in quotation marks "like this" to indicate direct quotes from your manuscript), or elaborate on this item by providing additional information not in the ms, or briefly explain why the item is not applicable/relevant for your study

Yes, "We therefore hypothesized that in the long-term, i.e., during 12 months, the stratified blended physiotherapy group patients would have improved self-management and adherence to prescribed LBP management strategies. These improvements could lead to an improvement in physical functioning and other clinical outcomes, which in turn could result in a reduction in societal and/or healthcare costs." AND "Therefore, the present study aimed to evaluate the long-term effectiveness on physical functioning and cost-effectiveness of stratified blended care (e-Exercise LBP) compared to face-to-face physiotherapy in patients with nonspecific LBP."

METHODS

3a) Description of trial design (such as parallel, factorial) including allocation ratio

Does your paper address CONSORT subitem 3a? *

Copy and paste relevant sections from the manuscript (include quotes in quotation marks "like this" to indicate direct quotes from your manuscript), or elaborate on this item by providing additional information not in the ms, or briefly explain why the item is not applicable/relevant for your study

Yes, "An economic evaluation was conducted alongside a prospective, multicenter cluster-randomized controlled trial (RCT)."

3b) Important changes to methods after trial commencement (such as eligibility criteria), with reasons

Does your paper address CONSORT subitem 3b? *

Copy and paste relevant sections from the manuscript (include quotes in quotation marks "like this" to indicate direct quotes from your manuscript), or elaborate on this item by providing additional information not in the ms, or briefly explain why the item is not applicable/relevant for your study

No, not applicable for this paper

3b-i) Bug fixes, Downtimes, Content Changes

Bug fixes, Downtimes, Content Changes: ehealth systems are often dynamic systems. A description of changes to methods therefore also includes important changes made on the intervention or comparator during the trial (e.g., major bug fixes or changes in the functionality or content) (5-iii) and other "unexpected events" that may have influenced study design such as staff changes, system failures/downtimes, etc. [2].

	1	2	3	4	5	
subitem not at all important	○	○	●	○	○	essential

Selectie wissen

Does your paper address subitem 3b-i?

Copy and paste relevant sections from the manuscript (include quotes in quotation marks "like this" to indicate direct quotes from your manuscript), or elaborate on this item by providing additional information not in the ms, or briefly explain why the item is not applicable/relevant for your study

Jouw antwoord

4a) Eligibility criteria for participants

Does your paper address CONSORT subitem 4a? *

Copy and paste relevant sections from the manuscript (include quotes in quotation marks "like this" to indicate direct quotes from your manuscript), or elaborate on this item by providing additional information not in the ms, or briefly explain why the item is not applicable/relevant for your study

Yes, "Patients were eligible if they requested physiotherapy treatment for nonspecific LBP, i.e., pain in the lumbosacral region (sometimes associated with pain radiating to the buttock or leg)30; were aged 18 years or older; possessed a smartphone or tablet (iOS or Android operating system) with access to the internet; and had sufficient command of the Dutch language. Physiotherapists informed potentially eligible patients about the study and informed the research team. The research team further informed the patient about the study, verified eligibility, and obtained written informed consent. Patients were excluded if they met any of the following criteria: a specific cause of LBP determined through medical imaging or a medical doctor; serious comorbidities (e.g., malignancy, stroke); and current pregnancy (because of the prevalence of pelvic girdle pain as a specific form of LBP)."

4a-i) Computer / Internet literacy

Computer / Internet literacy is often an implicit “de facto” eligibility criterion - this should be explicitly clarified.

	1	2	3	4	5	
subitem not at all important	○	○	◉	○	○	essential

Selectie wissen

Does your paper address subitem 4a-i?

Copy and paste relevant sections from the manuscript (include quotes in quotation marks "like this" to indicate direct quotes from your manuscript), or elaborate on this item by providing additional information not in the ms, or briefly explain why the item is not applicable/relevant for your study

Jouw antwoord

4a-ii) Open vs. closed, web-based vs. face-to-face assessments:

Open vs. closed, web-based vs. face-to-face assessments: Mention how participants were recruited (online vs. offline), e.g., from an open access website or from a clinic, and clarify if this was a purely web-based trial, or there were face-to-face components (as part of the intervention or for assessment), i.e., to what degree got the study team to know the participant. In online-only trials, clarify if participants were quasi-anonymous and whether having multiple identities was possible or whether technical or logistical measures (e.g., cookies, email confirmation, phone calls) were used to detect/prevent these.

	1	2	3	4	5	
subitem not at all important	○	○	◉	○	○	essential

Selectie wissen

Does your paper address subitem 4a-ii? *

Copy and paste relevant sections from the manuscript (include quotes in quotation marks "like this" to indicate direct quotes from your manuscript), or elaborate on this item by providing additional information not in the ms, or briefly explain why the item is not applicable/relevant for your study

Yes, "Patients were eligible if they requested physiotherapy treatment for nonspecific LBP, i.e., pain in the lumbosacral region (sometimes associated with pain radiating to the buttock or leg)30; were aged 18 years or older; possessed a smartphone or tablet (iOS or Android operating system) with access to the internet; and had sufficient command of the Dutch language. Physiotherapists informed potentially eligible patients about the study and informed the research team. The research team further informed the patient about the study, verified eligibility, and obtained written informed consent."

4a-iii) Information giving during recruitment

Information given during recruitment. Specify how participants were briefed for recruitment and in the informed consent procedures (e.g., publish the informed consent documentation as appendix, see also item X26), as this information may have an effect on user self-selection, user expectation and may also bias results.

	1	2	3	4	5	
subitem not at all important	○	○	◉	○	○	essential

Selectie wissen

Does your paper address subitem 4a-iii?

Copy and paste relevant sections from the manuscript (include quotes in quotation marks "like this" to indicate direct quotes from your manuscript), or elaborate on this item by providing additional information not in the ms, or briefly explain why the item is not applicable/relevant for your study

Yes, "Patients were eligible if they requested physiotherapy treatment for nonspecific LBP, i.e., pain in the lumbosacral region (sometimes associated with pain radiating to the buttock or leg)30; were aged 18 years or older; possessed a smartphone or tablet (iOS or Android operating system) with access to the internet; and had sufficient command of the Dutch language. Physiotherapists informed potentially eligible patients about the study and informed the research team. The research team further informed the patient about the study, verified eligibility, and obtained written informed consent."

4b) Settings and locations where the data were collected

Does your paper address CONSORT subitem 4b? *

Copy and paste relevant sections from the manuscript (include quotes in quotation marks "like this" to indicate direct quotes from your manuscript), or elaborate on this item by providing additional information not in the ms, or briefly explain why the item is not applicable/relevant for your study

Yes, "Primary and secondary clinical outcomes were assessed at baseline and at 3 and 12-month follow-ups using online questionnaires and an accelerometer. No financial incentives were offered to complete the measurements. Reminders were sent after seven and fourteen days." AND "Costs included intervention, other healthcare, informal care, absenteeism, presenteeism, and unpaid productivity costs due to nonspecific LBP. Costs were assessed at 3, 6, 9, and 12 months using 3-month retrospective self-reported cost questionnaires."

4b-i) Report if outcomes were (self-)assessed through online questionnaires

Clearly report if outcomes were (self-)assessed through online questionnaires (as common in web-based trials) or otherwise.

	1	2	3	4	5	
subitem not at all important	○	○	●	○	○	essential

Selectie wissen

Does your paper address subitem 4b-i? *

Copy and paste relevant sections from the manuscript (include quotes in quotation marks "like this" to indicate direct quotes from your manuscript), or elaborate on this item by providing additional information not in the ms, or briefly explain why the item is not applicable/relevant for your study

Yes, "Primary and secondary clinical outcomes were assessed at baseline and at 3 and 12-month follow-ups using online questionnaires and an accelerometer. No financial incentives were offered to complete the measurements. Reminders were sent after seven and fourteen days." AND "Costs included intervention, other healthcare, informal care, absenteeism, presenteeism, and unpaid productivity costs due to nonspecific LBP. Costs were assessed at 3, 6, 9, and 12 months using 3-month retrospective self-reported cost questionnaires."

4b-ii) Report how institutional affiliations are displayed

Report how institutional affiliations are displayed to potential participants [on ehealth media], as affiliations with prestigious hospitals or universities may affect volunteer rates, use, and reactions with regards to an intervention.(Not a required item – describe only if this may bias results)

	1	2	3	4	5	
subitem not at all important	○	○	◉	○	○	essential

Selectie wissen

Does your paper address subitem 4b-ii?

Copy and paste relevant sections from the manuscript (include quotes in quotation marks "like this" to indicate direct quotes from your manuscript), or elaborate on this item by providing additional information not in the ms, or briefly explain why the item is not applicable/relevant for your study

Jouw antwoord

5) The interventions for each group with sufficient details to allow replication, including how and when they were actually administered

5-i) Mention names, credential, affiliations of the developers, sponsors, and owners

Mention names, credential, affiliations of the developers, sponsors, and owners [6] (if authors/evaluators are owners or developer of the software, this needs to be declared in a “Conflict of interest” section or mentioned elsewhere in the manuscript).

	1	2	3	4	5	
subitem not at all important	○	○	◉	○	○	essential

Selectie wissen

Does your paper address subitem 5-i?

Copy and paste relevant sections from the manuscript (include quotes in quotation marks "like this" to indicate direct quotes from your manuscript), or elaborate on this item by providing additional information not in the ms, or briefly explain why the item is not applicable/relevant for your study

Jouw antwoord

5-ii) Describe the history/development process

Describe the history/development process of the application and previous formative evaluations (e.g., focus groups, usability testing), as these will have an impact on adoption/use rates and help with interpreting results.

	1	2	3	4	5	
subitem not at all important	○	○	◉	○	○	essential

Selectie wissen

Does your paper address subitem 5-ii?

Copy and paste relevant sections from the manuscript (include quotes in quotation marks "like this" to indicate direct quotes from your manuscript), or elaborate on this item by providing additional information not in the ms, or briefly explain why the item is not applicable/relevant for your study

Yes, "To investigate whether blended care for patients with nonspecific LBP can positively influence patients' self-management and adherence to prescribed management of LBP and consequently improve patients' physical functioning, we developed and evaluated the stratified blended physiotherapy intervention e-Exercise LBP27–29. In the short-term, i.e., after 3 months, e-Exercise LBP was not more effective than face-to-face physiotherapy in patients with nonspecific LBP in terms of physical functioning. However, patient self-reported adherence was significantly better among patients receiving e-Exercise LBP than among those receiving face-to-face physiotherapy only29."

5-iii) Revisions and updating

Revisions and updating. Clearly mention the date and/or version number of the application/intervention (and comparator, if applicable) evaluated, or describe whether the intervention underwent major changes during the evaluation process, or whether the development and/or content was "frozen" during the trial. Describe dynamic components such as news feeds or changing content which may have an impact on the replicability of the intervention (for unexpected events see item 3b).

	1	2	3	4	5	
subitem not at all important	○	○	◉	○	○	essential

Selectie wissen

Does your paper address subitem 5-iii?

Copy and paste relevant sections from the manuscript (include quotes in quotation marks "like this" to indicate direct quotes from your manuscript), or elaborate on this item by providing additional information not in the ms, or briefly explain why the item is not applicable/relevant for your study

Jouw antwoord

5-iv) Quality assurance methods

Provide information on quality assurance methods to ensure accuracy and quality of information provided [1], if applicable.

	1	2	3	4	5	
subitem not at all important	○	○	◉	○	○	essential

Selectie wissen

Does your paper address subitem 5-iv?

Copy and paste relevant sections from the manuscript (include quotes in quotation marks "like this" to indicate direct quotes from your manuscript), or elaborate on this item by providing additional information not in the ms, or briefly explain why the item is not applicable/relevant for your study

Jouw antwoord

5-v) Ensure replicability by publishing the source code, and/or providing screenshots/screen-capture video, and/or providing flowcharts of the algorithms used

Ensure replicability by publishing the source code, and/or providing screenshots/screen-capture video, and/or providing flowcharts of the algorithms used. Replicability (i.e., other researchers should in principle be able to replicate the study) is a hallmark of scientific reporting.

	1	2	3	4	5	
subitem not at all important	○	○	◉	○	○	essential

Selectie wissen

Does your paper address subitem 5-v?

Copy and paste relevant sections from the manuscript (include quotes in quotation marks "like this" to indicate direct quotes from your manuscript), or elaborate on this item by providing additional information not in the ms, or briefly explain why the item is not applicable/relevant for your study

Jouw antwoord

5-vi) Digital preservation

Digital preservation: Provide the URL of the application, but as the intervention is likely to change or disappear over the course of the years; also make sure the intervention is archived (Internet Archive, webcitation.org, and/or publishing the source code or screenshots/videos alongside the article). As pages behind login screens cannot be archived, consider creating demo pages which are accessible without login.

	1	2	3	4	5	
subitem not at all important	○	○	◉	○	○	essential

Selectie wissen

Does your paper address subitem 5-vi?

Copy and paste relevant sections from the manuscript (include quotes in quotation marks "like this" to indicate direct quotes from your manuscript), or elaborate on this item by providing additional information not in the ms, or briefly explain why the item is not applicable/relevant for your study

Jouw antwoord

5-vii) Access

Access: Describe how participants accessed the application, in what setting/context, if they had to pay (or were paid) or not, whether they had to be a member of specific group. If known, describe how participants obtained “access to the platform and Internet” [1]. To ensure access for editors/reviewers/readers, consider to provide a “backdoor” login account or demo mode for reviewers/readers to explore the application (also important for archiving purposes, see vi).

	1	2	3	4	5	
subitem not at all important	○	○	◉	○	○	essential

Selectie wissen

Does your paper address subitem 5-vii? *

Copy and paste relevant sections from the manuscript (include quotes in quotation marks "like this" to indicate direct quotes from your manuscript), or elaborate on this item by providing additional information not in the ms, or briefly explain why the item is not applicable/relevant for your study

Yes, "The content of both the face-to-face care and the smartphone application was tailored to the patients' individual needs and progress by the physiotherapists (Table 1)."

5-viii) Mode of delivery, features/functionalities/components of the intervention and comparator, and the theoretical framework

Describe mode of delivery, features/functionalities/components of the intervention and comparator, and the theoretical framework [6] used to design them (instructional strategy [1], behaviour change techniques, persuasive features, etc., see e.g., [7, 8] for terminology). This includes an in-depth description of the content (including where it is coming from and who developed it) [1]," whether [and how] it is tailored to individual circumstances and allows users to track their progress and receive feedback" [6]. This also includes a description of communication delivery channels and – if computer-mediated communication is a component – whether communication was synchronous or asynchronous [6]. It also includes information on presentation strategies [1], including page design principles, average amount of text on pages, presence of hyperlinks to other resources, etc. [1].

	1	2	3	4	5	
subitem not at all important	○	○	◉	○	○	essential

Selectie wissen

Does your paper address subitem 5-viii? *

Copy and paste relevant sections from the manuscript (include quotes in quotation marks "like this" to indicate direct quotes from your manuscript), or elaborate on this item by providing additional information not in the ms, or briefly explain why the item is not applicable/relevant for your study

Yes,
"Experimental: Stratified blended physiotherapy (e-Exercise LBP)
e-Exercise LBP is a stratified blended intervention in which a smartphone application is integrated into face-to-face physiotherapy treatment27,28. Both the content of the smartphone application and the face-to-face physiotherapy treatment are based on the recommendations of the guidelines for LBP of The Royal Dutch Society for Physiotherapy30. The duration and content of the stratified blended physiotherapy intervention was matched to the patients' risk for developing persistent LBP ('low', 'medium' or 'high') as assessed with the Keele STarT Back Screening Tool31. The smartphone application contains video-supported self-management information, video-supported exercises, and a goal-oriented physical activity module. The content of both the face-to-face care and the smartphone application was tailored to the patients' individual needs and progress by the physiotherapists (Table 1). Although physiotherapists were asked to treat according to the stratified blended physiotherapy protocol, they were allowed to deviate from the protocol according to their own clinical judgement. Print screens of the smartphone application are shown in Multimedia Appendix 3."

AND

"Control: Face-to-face physiotherapy
The face-to-face physiotherapy was in line with the LBP guidelines of The Royal Dutch Society for Physiotherapy30. The guidelines distinguish three different patient profiles based on the clinical course of recovery (i.e., normal recovery, abnormal recovery without predominant psychosocial factors, and abnormal recovery with predominant psychosocial factors) but do not use a specific tool to stratify care a priori. The content of the face-to-face physiotherapy was the same as that of the stratified blended care intervention, i.e., information, exercises, and recommendations regarding physical activity. However, no recommendations or restrictions were given regarding the number of face-to-face sessions. Physiotherapists were instructed to treat people without using any online applications to assure contrast between the two groups. The exact content of the therapy was left to the discretion of the physiotherapists and their clinical expertise."

5-ix) Describe use parameters

Describe use parameters (e.g., intended “doses” and optimal timing for use). Clarify what instructions or recommendations were given to the user, e.g., regarding timing, frequency, heaviness of use, if any, or was the intervention used ad libitum.

	1	2	3	4	5	
subitem not at all important	○	○	◉	○	○	essential

Selectie wissen

Does your paper address subitem 5-ix?

Copy and paste relevant sections from the manuscript (include quotes in quotation marks "like this" to indicate direct quotes from your manuscript), or elaborate on this item by providing additional information not in the ms, or briefly explain why the item is not applicable/relevant for your study

Jouw antwoord

5-x) Clarify the level of human involvement

Clarify the level of human involvement (care providers or health professionals, also technical assistance) in the e-intervention or as co-intervention (detail number and expertise of professionals involved, if any, as well as “type of assistance offered, the timing and frequency of the support, how it is initiated, and the medium by which the assistance is delivered”. It may be necessary to distinguish between the level of human involvement required for the trial, and the level of human involvement required for a routine application outside of a RCT setting (discuss under item 21 – generalizability).

	1	2	3	4	5	
subitem not at all important	○	○	◉	○	○	essential

Selectie wissen

Does your paper address subitem 5-x?

Copy and paste relevant sections from the manuscript (include quotes in quotation marks "like this" to indicate direct quotes from your manuscript), or elaborate on this item by providing additional information not in the ms, or briefly explain why the item is not applicable/relevant for your study

Jouw antwoord

5-xi) Report any prompts/reminders used

Report any prompts/reminders used: Clarify if there were prompts (letters, emails, phone calls, SMS) to use the application, what triggered them, frequency etc. It may be necessary to distinguish between the level of prompts/reminders required for the trial, and the level of prompts/reminders for a routine application outside of a RCT setting (discuss under item 21 – generalizability).

	1	2	3	4	5	
subitem not at all important	○	○	◉	○	○	essential

Selectie wissen

Does your paper address subitem 5-xi? *

Copy and paste relevant sections from the manuscript (include quotes in quotation marks "like this" to indicate direct quotes from your manuscript), or elaborate on this item by providing additional information not in the ms, or briefly explain why the item is not applicable/relevant for your study

No, is reported in more detail in our study protocol.

5-xii) Describe any co-interventions (incl. training/support)

Describe any co-interventions (incl. training/support): Clearly state any interventions that are provided in addition to the targeted eHealth intervention, as ehealth intervention may not be designed as stand-alone intervention. This includes training sessions and support [1]. It may be necessary to distinguish between the level of training required for the trial, and the level of training for a routine application outside of a RCT setting (discuss under item 21 – generalizability.

	1	2	3	4	5	
subitem not at all important	○	○	◉	○	○	essential

Selectie wissen

Does your paper address subitem 5-xii? *

Copy and paste relevant sections from the manuscript (include quotes in quotation marks "like this" to indicate direct quotes from your manuscript), or elaborate on this item by providing additional information not in the ms, or briefly explain why the item is not applicable/relevant for your study

Yes, "Physiotherapists from practices allocated to stratified blended physiotherapy received two 4-hour training sessions about e-Exercise LBP and the study procedures. Physiotherapists from practices allocated to face-to-face physiotherapy received one 4-hour training session in current best evidence practice and the study procedures." AND "Although physiotherapists were asked to treat according to the stratified blended physiotherapy protocol, they were allowed to deviate from the protocol according to their own clinical judgement."

6a) Completely defined pre-specified primary and secondary outcome measures, including how and when they were assessed

Does your paper address CONSORT subitem 6a? *

Copy and paste relevant sections from the manuscript (include quotes in quotation marks "like this" to indicate direct quotes from your manuscript), or elaborate on this item by providing additional information not in the ms, or briefly explain why the item is not applicable/relevant for your study

Yes,
"Outcome measures
Primary and secondary clinical outcomes were assessed at baseline and at 3 and 12-month follow-ups using online questionnaires and an accelerometer. No financial incentives were offered to complete the measurements. Reminders were sent after seven and fourteen days.

Primary outcome measures
For the effectiveness evaluation, the primary clinical outcome measure was physical functioning. Following the internationally accepted "Core Outcome Set" for research on patients with nonspecific LBP32, physical functioning was assessed with the Oswestry Disability Index (ODI), version 2.1a33. A higher ODI score indicates increased functional disability (range: 0-100).
For the economic evaluation, the primary outcomes were physical functioning and health-related quality of life. Health-related quality of life was assessed using the EuroQol-5D-5L (EQ-5D-5L)34,35. This questionnaire comprises five health dimensions (mobility, self-care, usual activities, pain/discomfort and anxiety/depression), all of which can be scored using five severity levels. With this, the instrument differentiates between 3,125 possible health states, which were converted into utility values (range: 0-1) using the Dutch tariff36. Quality-adjusted life years (QALYs) were calculated by multiplying the patients' utility values by their time spent in a certain health state using linear interpolation between measurement points37.

Secondary clinical outcome measures
Secondary clinical outcomes included average LBP intensity in the last week measured with an 11-point numeric rating scale (NRS)32,38; mean number of minutes per day spent in moderate to vigorous physical activity (MVPA) objectively measured using the Activ8 accelerometer (2M Engineering, Valkenswaard, The Netherlands)39; fear avoidance beliefs about physical activity and work measured using the Fear-Avoidance Beliefs Questionnaire (FABQ)40; pain catastrophizing measured by the Pain Catastrophizing Scale (PCS)41; self-efficacy measured using the General Self-efficacy Scale (GSE Scale)42,43; self-management ability assessed with the Dutch version of the short form Patient Activation Measure (PAM 13-Dutch)44; and patient self-reported adherence to prescribed home exercises measured with the Exercise Adherence Rating Scale (EARS)45. A detailed description of the secondary clinical outcome measures can be found elsewhere28,29.

Cost outcome measures
Costs included intervention, other healthcare, informal care, absenteeism, presenteeism, and unpaid productivity costs due to nonspecific LBP. Costs were assessed at 3, 6, 9, and 12 months using 3-month retrospective self-reported cost questionnaires. All costs were converted into Euros 2020 using consumer price indices46. Discounting of costs was not necessary due to the trial's 12-month follow-up.
Intervention costs were estimated based on the patients' total number of self-reported face-to-face physiotherapy and manual therapy sessions during the first three months of follow-up, valued using Dutch standard costs47. Intervention costs also comprised the cost per patient for the development, hosting, and maintenance of the stratified blended physiotherapy intervention. These costs were estimated by dividing the total development, hosting, and maintenance costs (i.e., €28,040) by the expected number of patients with

nonspecific LBP who would be eligible for the e-Exercise LBP study during the first 5 years after implementing it broadly (i.e., n=146,309)48 and an expected implementation rate of 10%. Hence, these costs were €0.19 per patient. Other healthcare costs included the cost of primary and secondary healthcare as well as medication use. Primary and secondary healthcare use were valued using Dutch standard costs47. If unavailable, prices according to professional organizations were used. Both prescribed and over-the-counter medication use were valued using unit prices derived from https://www.medicijnkosten.nl49. Informal care (i.e., care by family, friends, and other volunteers) was valued using a Dutch shadow price of €15.14/hour (in Euros 2020)47. Paid productivity losses comprised absenteeism (i.e., sickness absence) and presenteeism (i.e., reduced productivity while at work). Absenteeism was measured using a modified version of the IMTA Productivity Cost Questionnaire (iPCQ) and valued in accordance with the "Friction Cost Approach" (FCA) using gender-specific price weights50,51. The FCA assumes that costs are limited to the friction period (i.e., period needed to replace a sick worker = 85 days). Presenteeism was measured using the "Productivity and Disease Questionnaire" and valued using gender-specific price weights as well50–52. To assess unpaid productivity losses patients were asked to report the number of hours that they were not able to perform volunteer work and domestic and educational activities due to their nonspecific LBP, which were valued using the same Dutch shadow price of €15.14/hour47."

6a-i) Online questionnaires: describe if they were validated for online use and apply CHERRIES items to describe how the questionnaires were designed/deployed

If outcomes were obtained through online questionnaires, describe if they were validated for online use and apply CHERRIES items to describe how the questionnaires were designed/deployed [9].

	1	2	3	4	5	
subitem not at all important	○	○	◉	○	○	essential

Selectie wissen

Does your paper address subitem 6a-i?

Copy and paste relevant sections from manuscript text

Jouw antwoord

6a-ii) Describe whether and how “use” (including intensity of use/dosage) was defined/measured/monitored

Describe whether and how “use” (including intensity of use/dosage) was defined/measured/monitored (logins, logfile analysis, etc.). Use/adoption metrics are important process outcomes that should be reported in any ehealth trial.

	1	2	3	4	5	
subitem not at all important	○	○	◉	○	○	essential

Selectie wissen

Does your paper address subitem 6a-ii?

Copy and paste relevant sections from manuscript text

Jouw antwoord

6a-iii) Describe whether, how, and when qualitative feedback from participants was obtained

Describe whether, how, and when qualitative feedback from participants was obtained (e.g., through emails, feedback forms, interviews, focus groups).

	1	2	3	4	5	
subitem not at all important	○	○	◉	○	○	essential

Selectie wissen

Does your paper address subitem 6a-iii?

Copy and paste relevant sections from manuscript text

Jouw antwoord

6b) Any changes to trial outcomes after the trial commenced, with reasons

Does your paper address CONSORT subitem 6b? *

Copy and paste relevant sections from the manuscript (include quotes in quotation marks "like this" to indicate direct quotes from your manuscript), or elaborate on this item by providing additional information not in the ms, or briefly explain why the item is not applicable/relevant for your study

No, not applicbale for this paper

7a) How sample size was determined

NPT: When applicable, details of whether and how the clustering by care provides or centers was addressed

7a-i) Describe whether and how expected attrition was taken into account when calculating the sample size

Describe whether and how expected attrition was taken into account when calculating the sample size.

	1	2	3	4	5	
subitem not at all important	○	○	◉	○	○	essential

Selectie wissen

Does your paper address subitem 7a-i?

Copy and paste relevant sections from manuscript title (include quotes in quotation marks "like this" to indicate direct quotes from your manuscript), or elaborate on this item by providing additional information not in the ms, or briefly explain why the item is not applicable/relevant for your study

Yes, "Sample size calculations were based upon the recommendations of Campbell et al. for cluster randomized trials58. To detect clinically relevant mean differences between groups at the 12-month follow-up, a difference of >6 points in physical functioning (ODI) and a standard deviation of 14.5 were used59–61. In addition, repeated measures of the primary outcome during follow-up were taken into account, and an intraclass correlation coefficient of 0.05 was used. For the repeated measures of physical functioning, a correlation of 0.5 was estimated between baseline and follow-up measurements until the 12-month follow-up62. Based on these assumptions (power 80%, α=0.05) and an average cluster size of 5, 165 patients were needed. With an expected dropout rate of 20%, a total of 207 participating patients (n=104 per arm) were needed."

7b) When applicable, explanation of any interim analyses and stopping guidelines

Does your paper address CONSORT subitem 7b? *

Copy and paste relevant sections from the manuscript (include quotes in quotation marks "like this" to indicate direct quotes from your manuscript), or elaborate on this item by providing additional information not in the ms, or briefly explain why the item is not applicable/relevant for your study

No, not applicbale for this paper

8a) Method used to generate the random allocation sequence
NPT: When applicable, how care providers were allocated to each trial group

Does your paper address CONSORT subitem 8a? *

Copy and paste relevant sections from the manuscript (include quotes in quotation marks "like this" to indicate direct quotes from your manuscript), or elaborate on this item by providing additional information not in the ms, or briefly explain why the item is not applicable/relevant for your study

Yes, "A total of 58 Dutch primary care physiotherapy practices with 122 physiotherapists were randomized on the practice level by an independent researcher according to a 1:1 allocation ratio using a computer-generated, a priori created, random sequence table."

8b) Type of randomisation; details of any restriction (such as blocking and block size)

Does your paper address CONSORT subitem 8b? *

Copy and paste relevant sections from the manuscript (include quotes in quotation marks "like this" to indicate direct quotes from your manuscript), or elaborate on this item by providing additional information not in the ms, or briefly explain why the item is not applicable/relevant for your study

Yes, "A total of 58 Dutch primary care physiotherapy practices with 122 physiotherapists were randomized on the practice level by an independent researcher according to a 1:1 allocation ratio using a computer-generated, a priori created, random sequence table."

9) Mechanism used to implement the random allocation sequence (such as sequentially numbered containers), describing any steps taken to conceal the sequence until interventions were assigned

Does your paper address CONSORT subitem 9? *

Copy and paste relevant sections from the manuscript (include quotes in quotation marks "like this" to indicate direct quotes from your manuscript), or elaborate on this item by providing additional information not in the ms, or briefly explain why the item is not applicable/relevant for your study

Yes, "A total of 58 Dutch primary care physiotherapy practices with 122 physiotherapists were randomized on the practice level by an independent researcher according to a 1:1 allocation ratio using a computer-generated, a priori created, random sequence table."

10) Who generated the random allocation sequence, who enrolled participants, and who assigned participants to interventions

Does your paper address CONSORT subitem 10? *

Copy and paste relevant sections from the manuscript (include quotes in quotation marks "like this" to indicate direct quotes from your manuscript), or elaborate on this item by providing additional information not in the ms, or briefly explain why the item is not applicable/relevant for your study

Yes, "A total of 58 Dutch primary care physiotherapy practices with 122 physiotherapists were randomized on the practice level by an independent researcher according to a 1:1 allocation ratio using a computer-generated, a priori created, random sequence table. Half of the practices (n=29) were instructed to treat their patients with nonspecific LBP according to the stratified blended physiotherapy (e-Exercise LBP) protocol. The other half (n=29) treated their patients with face-to-face care following the recommendations of the guidelines for LBP of The Royal Dutch Society for Physiotherapy30." AND "Physiotherapists informed potentially eligible patients about the study and informed the research team. The research team further informed the patient about the study, verified eligibility, and obtained written informed consent."

11a) If done, who was blinded after assignment to interventions (for example, participants, care providers, those assessing outcomes) and how

NPT: Whether or not administering co-interventions were blinded to group assignment

11a-i) Specify who was blinded, and who wasn't

Specify who was blinded, and who wasn't. Usually, in web-based trials it is not possible to blind the participants [1, 3] (this should be clearly acknowledged), but it may be possible to blind outcome assessors, those doing data analysis or those administering co-interventions (if any).

	1	2	3	4	5	
subitem not at all important	○	○	◉	○	○	essential

Selectie wissen

Does your paper address subitem 11a-i? *

Copy and paste relevant sections from the manuscript (include quotes in quotation marks "like this" to indicate direct quotes from your manuscript), or elaborate on this item by providing additional information not in the ms, or briefly explain why the item is not applicable/relevant for your study

Yes, but not explicitely stated, "A total of 58 Dutch primary care physiotherapy practices with 122 physiotherapists were randomized on the practice level by an independent researcher according to a 1:1 allocation ratio using a computer-generated, a priori created, random sequence table. Half of the practices (n=29) were instructed to treat their patients with nonspecific LBP according to the stratified blended physiotherapy (e-Exercise LBP) protocol. The other half (n=29) treated their patients with face-to-face care following the recommendations of the guidelines for LBP of The Royal Dutch Society for Physiotherapy30."

11a-ii) Discuss e.g., whether participants knew which intervention was the "intervention of interest" and which one was the "comparator"

Informed consent procedures (4a-ii) can create biases and certain expectations - discuss e.g., whether participants knew which intervention was the "intervention of interest" and which one was the "comparator".

	1	2	3	4	5	
subitem not at all important	○	○	○	○	○	essential

Does your paper address subitem 11a-ii?

Copy and paste relevant sections from the manuscript (include quotes in quotation marks "like this" to indicate direct quotes from your manuscript), or elaborate on this item by providing additional information not in the ms, or briefly explain why the item is not applicable/relevant for your study

Jouw antwoord

11b) If relevant, description of the similarity of interventions

(this item is usually not relevant for ehealth trials as it refers to similarity of a placebo or sham intervention to a active medication/intervention)

Does your paper address CONSORT subitem 11b? *

Copy and paste relevant sections from the manuscript (include quotes in quotation marks "like this" to indicate direct quotes from your manuscript), or elaborate on this item by providing additional information not in the ms, or briefly explain why the item is not applicable/relevant for your study

Yes, "Both the content of the smartphone application and the face-to-face physiotherapy treatment are based on the recommendations of the guidelines for LBP of The Royal Dutch Society for Physiotherapy30."

12a) Statistical methods used to compare groups for primary and secondary outcomes

NPT: When applicable, details of whether and how the clustering by care providers or centers was addressed

Does your paper address CONSORT subitem 12a? *

Copy and paste relevant sections from the manuscript (include quotes in quotation marks "like this" to indicate direct quotes from your manuscript), or elaborate on this item by providing additional information not in the ms, or briefly explain why the item is not applicable/relevant for your study

Yes, "Analysis of effectiveness
The effectiveness of stratified blended physiotherapy compared to face-to-face physiotherapy for the primary and secondary clinical outcomes was estimated using linear mixed models (LMMs). A two-level structure was used, existing of repeated measurements (level 1), nested within patients (level 2). The necessity of using additional levels in the random effects model to control for the clustering of patients within physiotherapy practices and individual physiotherapists was checked using log likelihood ratios56. Overall mean differences for the complete duration of follow-up, as well as mean differences per time point, were estimated between stratified blended physiotherapy and face-to-face physiotherapy. Regression coefficients with 95% confidence intervals (CIs) were used to signify the differences between stratified blended physiotherapy and face-to-face physiotherapy. Analyses were adjusted for baseline values of clinical outcome measures (e.g., utility score and physical functioning) and variables with a substantial difference at baseline that changed the regression coefficient for the between-group estimate by ≥10% (i.e., duration of LBP complaints).

Analysis of cost-effectiveness
As indicated above, an economic evaluation was performed from both societal and healthcare perspectives. When the societal perspective was applied, all costs were included. When the healthcare perspective was applied, only costs accruing to the formal Dutch healthcare sector were included.
Mean between-group cost differences were calculated for total and disaggregated costs using ordinary least squares (OLS) regression analyses. Seemingly unrelated regression (SUR) analyses were performed to estimate total cost and effect differences (i.e., ΔC and ΔE), while adjusting for baseline values and confounders and taking into account the possible correlation between costs and effects. Variables were considered confounders if they differed considerably at baseline between groups and/or changed the regression coefficient by more than 10%. For effects, the duration of LBP complaints was a confounder. For costs, the confounders were employment status (societal perspective) and the duration of complaints (healthcare perspective). Incremental cost-effectiveness ratios (ICERs) were calculated by dividing the adjusted differences in total costs by the adjusted differences in effects (i.e., ΔC/ΔE). Bias-corrected and accelerated bootstrapping with 5000 replications was used to estimate the uncertainty surrounding the cost differences and ICERs. Uncertainty surrounding the ICERs was graphically illustrated by plotting bootstrapped cost-effect pairs on cost-effectiveness planes. Cost-effectiveness acceptability curves (CEACs) were constructed to indicate the probability of stratified blended physiotherapy being cost-effective in comparison to face-to-face physiotherapy at different values of willingness-to-pay37. In the Netherlands, threshold values for willingness-to-pay of €10,000 to €80,000 per QALY are commonly used for societal perspective analyses57. For physical functioning, such threshold values are currently lacking."

12a-i) Imputation techniques to deal with attrition / missing values

Imputation techniques to deal with attrition / missing values: Not all participants will use the intervention/comparator as intended and attrition is typically high in ehealth trials. Specify how participants who did not use the application or dropped out from the trial were treated in the statistical analysis (a complete case analysis is strongly discouraged, and simple imputation techniques such as LOCF may also be problematic [4]).

	1	2	3	4	5	
subitem not at all important	○	○	◉	○	○	essential

Selectie wissen

Does your paper address subitem 12a-i? *

Copy and paste relevant sections from the manuscript (include quotes in quotation marks "like this" to indicate direct quotes from your manuscript), or elaborate on this item by providing additional information not in the ms, or briefly explain why the item is not applicable/relevant for your study

Yes, "Statistical analyses were performed according to the intention-to-treat principle. Descriptive statistics were used to explore between-group baseline comparability and to describe patients' general characteristics. Using multivariate imputation by chained equations (MICE) with predictive mean matching, 10 complete datasets were created (loss-of-efficiency <5%)55. The imputation model consisted of variables that differed between groups at baseline, variables that were related to the "missingness" of data, variables associated with the outcome, and all available baseline and follow-up costs and clinical outcome measures. Then, each imputed dataset was analyzed separately as specified below. Pooled estimates were calculated using Rubin's rules, incorporating both within-imputation variability (i.e., uncertainty about the results from one imputed dataset) and between-imputation variability (i.e., reflecting the uncertainty due to missing information)55. Analysis of effectiveness and cost-effectiveness were performed using STATA Corp 13.0."

12b) Methods for additional analyses, such as subgroup analyses and adjusted analyses

Does your paper address CONSORT subitem 12b? *

Copy and paste relevant sections from the manuscript (include quotes in quotation marks "like this" to indicate direct quotes from your manuscript), or elaborate on this item by providing additional information not in the ms, or briefly explain why the item is not applicable/relevant for your study

Yes, "Three sensitivity analyses were performed as part of the economic evaluation. In the first sensitivity analysis, only data from complete cases on the primary clinical outcome and cost outcome measures were included. In a second sensitivity analysis, absenteeism costs were estimated using the human capital approach (HCA), assuming that productivity losses are generated during the entire duration of absence. In the third sensitivity analysis, the analysis was performed per risk group for developing persistent LBP (low, medium and high) separately, since this proved to be an effect modifier of the between-group differences between stratified blended physiotherapy and face-to-face physiotherapy in the short-term29."

X26) REB/IRB Approval and Ethical Considerations [recommended as subheading under "Methods"] (not a CONSORT item)

X26-i) Comment on ethics committee approval

	1	2	3	4	5	
subitem not at all important	○	○	◉	○	○	essential

Selectie wissen

Does your paper address subitem X26-i?

Copy and paste relevant sections from the manuscript (include quotes in quotation marks "like this" to indicate direct quotes from your manuscript), or elaborate on this item by providing additional information not in the ms, or briefly explain why the item is not applicable/relevant for your study

Jouw antwoord

x26-ii) Outline informed consent procedures

Outline informed consent procedures e.g., if consent was obtained offline or online (how? Checkbox, etc.?), and what information was provided (see 4a-ii). See [6] for some items to be included in informed consent documents.

	1	2	3	4	5	
subitem not at all important	○	○	◉	○	○	essential

Selectie wissen

Does your paper address subitem X26-ii?

Copy and paste relevant sections from the manuscript (include quotes in quotation marks "like this" to indicate direct quotes from your manuscript), or elaborate on this item by providing additional information not in the ms, or briefly explain why the item is not applicable/relevant for your study

Yes, "The research team further informed the patient about the study, verified eligibility, and obtained written informed consent."

X26-iii) Safety and security procedures

Safety and security procedures, incl. privacy considerations, and any steps taken to reduce the likelihood or detection of harm (e.g., education and training, availability of a hotline)

	1	2	3	4	5	
subitem not at all important	○	○	◉	○	○	essential

Selectie wissen

Does your paper address subitem X26-iii?

Copy and paste relevant sections from the manuscript (include quotes in quotation marks "like this" to indicate direct quotes from your manuscript), or elaborate on this item by providing additional information not in the ms, or briefly explain why the item is not applicable/relevant for your study

Jouw antwoord

RESULTS

13a) For each group, the numbers of participants who were randomly assigned, received intended treatment, and were analysed for the primary outcome
NPT: The number of care providers or centers performing the intervention in each group and the number of patients treated by each care provider in each center

Does your paper address CONSORT subitem 13a? *

Copy and paste relevant sections from the manuscript (include quotes in quotation marks "like this" to indicate direct quotes from your manuscript), or elaborate on this item by providing additional information not in the ms, or briefly explain why the item is not applicable/relevant for your study

Yes, "Flow of participants, therapists, and centers through the study
In total, 208 eligible patients participated; 104 were allocated to the stratified blended physiotherapy group and 104 were allocated to the face-to-face physiotherapy group (Figure 1). Complete data on all primary clinical and cost outcome measures were obtained from 171 (82.2%) patients. Four patients (n=2 stratified blended physiotherapy; n=2 face-to-face physiotherapy) were excluded from all analyses since they were diagnosed with specific LBP immediately after inclusion, and hence did not meet the in- and exclusion criteria anymore. At baseline, the stratified blended physiotherapy group consisted of more males, more patients with a low level of education, and more patients with a duration of LBP >12 months than the face-to-face physiotherapy group. No other relevant differences in baseline characteristics were seen between groups (Table 2)."

13b) For each group, losses and exclusions after randomisation, together with reasons

Does your paper address CONSORT subitem 13b? (NOTE: Preferably, this is shown in a CONSORT flow diagram) *

Copy and paste relevant sections from the manuscript (include quotes in quotation marks "like this" to indicate direct quotes from your manuscript), or elaborate on this item by providing additional information not in the ms, or briefly explain why the item is not applicable/relevant for your study

Yes, "Flow of participants, therapists, and centers through the study
In total, 208 eligible patients participated; 104 were allocated to the stratified blended physiotherapy group and 104 were allocated to the face-to-face physiotherapy group (Figure 1). Complete data on all primary clinical and cost outcome measures were obtained from 171 (82.2%) patients. Four patients (n=2 stratified blended physiotherapy; n=2 face-to-face physiotherapy) were excluded from all analyses since they were diagnosed with specific LBP immediately after inclusion, and hence did not meet the in- and exclusion criteria anymore. At baseline, the stratified blended physiotherapy group consisted of more males, more patients with a low level of education, and more patients with a duration of LBP >12 months than the face-to-face physiotherapy group. No other relevant differences in baseline characteristics were seen between groups (Table 2)."

13b-i) Attrition diagram

Strongly recommended: An attrition diagram (e.g., proportion of participants still logging in or using the intervention/comparator in each group plotted over time, similar to a survival curve) or other figures or tables demonstrating usage/dose/engagement.

	1	2	3	4	5	
subitem not at all important	○	○	●	○	○	essential

Selectie wissen

Does your paper address subitem 13b-i?

Copy and paste relevant sections from the manuscript or cite the figure number if applicable (include quotes in quotation marks "like this" to indicate direct quotes from your manuscript), or elaborate on this item by providing additional information not in the ms, or briefly explain why the item is not applicable/relevant for your study

Jouw antwoord

14a) Dates defining the periods of recruitment and follow-up

Does your paper address CONSORT subitem 14a? *

Copy and paste relevant sections from the manuscript (include quotes in quotation marks "like this" to indicate direct quotes from your manuscript), or elaborate on this item by providing additional information not in the ms, or briefly explain why the item is not applicable/relevant for your study

Yes, "Enrollment of patients lasted from June 2018 until December 2019, and follow-up lasted 24 months. This paper evaluates the 12-month effectiveness and cost-effectiveness."

14a-i) Indicate if critical “secular events” fell into the study period

Indicate if critical “secular events” fell into the study period, e.g., significant changes in Internet resources available or “changes in computer hardware or Internet delivery resources”

	1	2	3	4	5	
subitem not at all important	○	○	◉	○	○	essential

Selectie wissen

Does your paper address subitem 14a-i?

Copy and paste relevant sections from the manuscript (include quotes in quotation marks "like this" to indicate direct quotes from your manuscript), or elaborate on this item by providing additional information not in the ms, or briefly explain why the item is not applicable/relevant for your study

Jouw antwoord

14b) Why the trial ended or was stopped (early)

Does your paper address CONSORT subitem 14b? *

Copy and paste relevant sections from the manuscript (include quotes in quotation marks "like this" to indicate direct quotes from your manuscript), or elaborate on this item by providing additional information not in the ms, or briefly explain why the item is not applicable/relevant for your study

No, not applicable. Desired sample size was reached.

15) A table showing baseline demographic and clinical characteristics for each group

NPT: When applicable, a description of care providers (case volume, qualification, expertise, etc.) and centers (volume) in each group

Does your paper address CONSORT subitem 15? *

Copy and paste relevant sections from the manuscript (include quotes in quotation marks "like this" to indicate direct quotes from your manuscript), or elaborate on this item by providing additional information not in the ms, or briefly explain why the item is not applicable/relevant for your study

Yes, Table 2 contains this.

15-i) Report demographics associated with digital divide issues

In ehealth trials it is particularly important to report demographics associated with digital divide issues, such as age, education, gender, social-economic status, computer/Internet/ehealth literacy of the participants, if known.

	1	2	3	4	5	
subitem not at all important	○	○	◉	○	○	essential

Selectie wissen

Does your paper address subitem 15-i? *

Copy and paste relevant sections from the manuscript (include quotes in quotation marks "like this" to indicate direct quotes from your manuscript), or elaborate on this item by providing additional information not in the ms, or briefly explain why the item is not applicable/relevant for your study

Yes, partly. Information can be found in Table 2.

16) For each group, number of participants (denominator) included in each analysis and whether the analysis was by original assigned groups

16-i) Report multiple “denominators” and provide definitions

Report multiple “denominators” and provide definitions: Report N’s (and effect sizes) “across a range of study participation [and use] thresholds” [1], e.g., N exposed, N consented, N used more than x times, N used more than y weeks, N participants “used” the intervention/comparator at specific pre-defined time points of interest (in absolute and relative numbers per group). Always clearly define “use” of the intervention.

	1	2	3	4	5	
subitem not at all important	○	○	◉	○	○	essential

Selectie wissen

Does your paper address subitem 16-i? *

Copy and paste relevant sections from the manuscript (include quotes in quotation marks "like this" to indicate direct quotes from your manuscript), or elaborate on this item by providing additional information not in the ms, or briefly explain why the item is not applicable/relevant for your study

Yes, Table 3 and Table 4 and Table 5.

16-ii) Primary analysis should be intent-to-treat

Primary analysis should be intent-to-treat, secondary analyses could include comparing only “users”, with the appropriate caveats that this is no longer a randomized sample (see 18-i).

	1	2	3	4	5	
subitem not at all important	○	○	◉	○	○	essential

Selectie wissen

Does your paper address subitem 16-ii?

Copy and paste relevant sections from the manuscript (include quotes in quotation marks "like this" to indicate direct quotes from your manuscript), or elaborate on this item by providing additional information not in the ms, or briefly explain why the item is not applicable/relevant for your study

Jouw antwoord

17a) For each primary and secondary outcome, results for each group, and the estimated effect size and its precision (such as 95% confidence interval)

Does your paper address CONSORT subitem 17a? *

Copy and paste relevant sections from the manuscript (include quotes in quotation marks "like this" to indicate direct quotes from your manuscript), or elaborate on this item by providing additional information not in the ms, or briefly explain why the item is not applicable/relevant for your study

Yes, "Both interventions were associated with improved clinical outcomes from baseline to 12-months follow-up (within-group differences are presented in Multimedia Appendix 4). From a clinical perspective, there was neither a clinically relevant, nor a statistically significant adjusted between-group difference over 12 months in the primary outcome of physical functioning (mean difference (MD) -1.1; 95% CI, -3.9 to 1.7). Per time point, adjusted between-group differences in physical functioning were neither clinically relevant, nor statistically significant. For the secondary clinical outcomes, a statistically significant adjusted between-group difference over 12 months, and per time point, was found in favor of stratified blended physiotherapy for fear avoidance beliefs, i.e. Overall MD -4.3; 95% CI, -7.3 to -1.3, 3-month MD -3.9; 95% CI, -7.5 to -0.4, and 12-month MD -4.7; 95% CI, -8.5 to -0.9, respectively. Also, at the 3-month time point, a statistically significant adjusted between-group difference was found in favor of stratified blended physiotherapy for patients' self-reported adherence to prescribed home exercises (MD 0.8; 95% CI, 0.1 to 1.6). Overall differences in secondary clinical outcomes and differences per time point were not considered clinically relevant (Table 3)."

AND

"Societal perspective
The ICER for QALYs was 49,159, indicating that – on average – stratified blended physiotherapy was associated with an additional cost of €49,159 per QALY gained compared with face-to-face physiotherapy (Table 5, Figure 2a). The CEAC indicated that if society is not willing to pay anything per QALY gained, the probability of stratified blended physiotherapy being cost-effective compared to face-to-face physiotherapy is 0.23 (Figure 3a). This probability increased to a maximum of 0.50 at a willingness to pay of €50,000/QALY.
For physical functioning, the ICER was -614. This indicates that stratified blended physiotherapy was – on average – associated with a societal cost of €614 per 1-point improvement on the ODI compared with face-to-face physiotherapy (Table 5, Figure 2b). Please note that a lower ODI score indicates an improved level of physical functioning. The CEAC shows that if decision-makers are not willing to pay anything per 1-point improvement on the ODI, the probability of stratified blended physiotherapy being cost-effective compared to face-to-face physiotherapy was 0.23 (Figure 3b). This probability increased to 0.63 at a willingness to pay of €1,000/point improvement and to 0.79 at a willingness to pay of €10,000/point improvement.

Healthcare perspective
The ICER for QALYs was 2,239, indicating that stratified blended physiotherapy was – on average – associated with an additional cost of €2,239 per QALY gained compared with face-to-face physiotherapy (Table 5, Figure 2c). The CEAC indicated that if the healthcare system is not willing to pay anything per QALY gained, the probability of stratified blended physiotherapy being cost-effective compared to face-to-face physiotherapy is 0.27 (Figure 3c). This probability gradually increased to a maximum of 0.75 at a willingness to pay of €10,000/QALY.
For physical functioning, the ICER was -28. This indicates that stratified blended physiotherapy was – on average – associated with a healthcare cost of €28 per 1-point improvement on the ODI compared with face-to-face physiotherapy (Table 5, Figure 2d). The

CEAC shows that if decision-makers are not willing to pay anything per 1-point improvement on the ODI, the probability of stratified blended physiotherapy being cost-effective compared to face-to-face physiotherapy is 0.27 (Figure 3d). This probability increased to 0.81 at a willingness to pay of €1,000/point improvement and remained the same at a higher willingness to pay."

AND Table 3 and Table 4 and Table 5.

17a-i) Presentation of process outcomes such as metrics of use and intensity of use

In addition to primary/secondary (clinical) outcomes, the presentation of process outcomes such as metrics of use and intensity of use (dose, exposure) and their operational definitions is critical. This does not only refer to metrics of attrition (13-b) (often a binary variable), but also to more continuous exposure metrics such as "average session length". These must be accompanied by a technical description how a metric like a "session" is defined (e.g., timeout after idle time) [1] (report under item 6a).

	1	2	3	4	5	
subitem not at all important	○	○	◉	○	○	essential

Selectie wissen

Does your paper address subitem 17a-i?

Copy and paste relevant sections from the manuscript (include quotes in quotation marks "like this" to indicate direct quotes from your manuscript), or elaborate on this item by providing additional information not in the ms, or briefly explain why the item is not applicable/relevant for your study

Jouw antwoord

17b) For binary outcomes, presentation of both absolute and relative effect sizes is recommended

Does your paper address CONSORT subitem 17b? *

Copy and paste relevant sections from the manuscript (include quotes in quotation marks "like this" to indicate direct quotes from your manuscript), or elaborate on this item by providing additional information not in the ms, or briefly explain why the item is not applicable/relevant for your study

No, not applicable for this paper.

18) Results of any other analyses performed, including subgroup analyses and adjusted analyses, distinguishing pre-specified from exploratory

Does your paper address CONSORT subitem 18? *

Copy and paste relevant sections from the manuscript (include quotes in quotation marks "like this" to indicate direct quotes from your manuscript), or elaborate on this item by providing additional information not in the ms, or briefly explain why the item is not applicable/relevant for your study

Yes, "The direction and magnitude of the differences in costs and effects between the stratified blended physiotherapy group and the face-to-face physiotherapy group as estimated in the sensitivity analyses were not completely in line with those estimated in the main analysis. In particular, when analyzing complete cases only (sensitivity analysis 1), cost differences between the stratified blended physiotherapy group and the face-to-face physiotherapy group were found to be in favor of the stratified blended physiotherapy group, whereas when missing values were imputed (main analysis), these cost differences were in favor of the face-to-face physiotherapy group. This resulted in slightly different CEACs than those obtained in the main analysis. The results of sensitivity analysis 3 showed that the cost difference between the stratified blended physiotherapy group and the face-to-face physiotherapy group increased with a higher risk of developing persistent LBP. That is, for the low-, medium- and high-risk groups, the differences in costs for the societal perspective (QALYs) were €46, €1,124 and €5,225, respectively. In line with the main analysis, however, stratified blended physiotherapy did not seem to be cost-effective in any of the sensitivity analyses (Table 5)."

18-i) Subgroup analysis of comparing only users

A subgroup analysis of comparing only users is not uncommon in ehealth trials, but if done, it must be stressed that this is a self-selected sample and no longer an unbiased sample from a randomized trial (see 16-iii).

	1	2	3	4	5	
subitem not at all important	○	○	◉	○	○	essential

Selectie wissen

Does your paper address subitem 18-i?

Copy and paste relevant sections from the manuscript (include quotes in quotation marks "like this" to indicate direct quotes from your manuscript), or elaborate on this item by providing additional information not in the ms, or briefly explain why the item is not applicable/relevant for your study

Jouw antwoord

19) All important harms or unintended effects in each group
(for specific guidance see CONSORT for harms)

Does your paper address CONSORT subitem 19? *

Copy and paste relevant sections from the manuscript (include quotes in quotation marks "like this" to indicate direct quotes from your manuscript), or elaborate on this item by providing additional information not in the ms, or briefly explain why the item is not applicable/relevant for your study

No, not applicable for this paper

19-i) Include privacy breaches, technical problems

Include privacy breaches, technical problems. This does not only include physical “harm” to participants, but also incidents such as perceived or real privacy breaches [1], technical problems, and other unexpected/unintended incidents. “Unintended effects” also includes unintended positive effects [2].

	1	2	3	4	5	
subitem not at all important	○	○	◉	○	○	essential

Selectie wissen

Does your paper address subitem 19-i?

Copy and paste relevant sections from the manuscript (include quotes in quotation marks "like this" to indicate direct quotes from your manuscript), or elaborate on this item by providing additional information not in the ms, or briefly explain why the item is not applicable/relevant for your study

Jouw antwoord

19-ii) Include qualitative feedback from participants or observations from staff/researchers

Include qualitative feedback from participants or observations from staff/researchers, if available, on strengths and shortcomings of the application, especially if they point to unintended/unexpected effects or uses. This includes (if available) reasons for why people did or did not use the application as intended by the developers.

	1	2	3	4	5	
subitem not at all important	○	○	◉	○	○	essential

Selectie wissen

Does your paper address subitem 19-ii?

Copy and paste relevant sections from the manuscript (include quotes in quotation marks "like this" to indicate direct quotes from your manuscript), or elaborate on this item by providing additional information not in the ms, or briefly explain why the item is not applicable/relevant for your study

Jouw antwoord

DISCUSSION

22) Interpretation consistent with results, balancing benefits and harms, and considering other relevant evidence

NPT: In addition, take into account the choice of the comparator, lack of or partial blinding, and unequal expertise of care providers or centers in each group

22-i) Restate study questions and summarize the answers suggested by the data, starting with primary outcomes and process outcomes (use)

Restate study questions and summarize the answers suggested by the data, starting with primary outcomes and process outcomes (use).

	1	2	3	4	5	
subitem not at all important	○	○	◉	○	○	essential

Selectie wissen

Does your paper address subitem 22-i? *

Copy and paste relevant sections from the manuscript (include quotes in quotation marks "like this" to indicate direct quotes from your manuscript), or elaborate on this item by providing additional information not in the ms, or briefly explain why the item is not applicable/relevant for your study

Yes, "This study evaluated the long-term effectiveness and cost-effectiveness of the stratified blended physiotherapy intervention e-Exercise LBP in comparison to face-to-face physiotherapy in patients with nonspecific LBP. Both interventions were associated with improved clinical outcomes from baseline to 12-months follow-up, but the study results showed neither a clinically relevant, nor a statistically significant between-group difference in physical functioning. Over 12 months, and for each time point, only fear avoidance beliefs improved significantly more in patients who were allocated to the e-Exercise LBP group. At 3-months, patients who were allocated to the e-Exercise LBP group reported a better adherence to prescribed home exercises. However, the overall between-group difference and the differences in improvement per time point in both fear avoidance beliefs and self-reported adherence to prescribed home exercises were not considered clinically relevant. As for the intervention's cost-effectiveness, from both the societal and healthcare perspectives, a considerable amount of money must be paid per additional QALY or 1-point improvement in physical functioning to reach a relatively low to moderate probability of e-Exercise LBP being cost-effective compared to face-to-face physiotherapy. To illustrate, e-Exercise LBP had a low probability (i.e., 0.29 and 0.60) of cost-effectiveness at the upper and lower bounds of the informal Dutch willingness-to-pay threshold for QALYs (i.e., €10,000 to €80,000 per QALY). For the healthcare perspective and the outcome of physical functioning, willingness-to-pay thresholds are lacking. However, we consider the maximum probability of e-Exercise LBP being cost-effective compared to face-to-face physiotherapy for both outcomes to be moderate at best (i.e., <0.81). Hence, from both societal and healthcare perspectives, e-Exercise LBP does not seem to be cost-effective compared to face-to-face physiotherapy among patients with nonspecific LBP. Between-group differences in costs and effects as estimated in the sensitivity analyses were not completely in line with our main analysis. However, conclusions of the sensitivity analysis confirmed our main analysis."

22-ii) Highlight unanswered new questions, suggest future research

Highlight unanswered new questions, suggest future research.

	1	2	3	4	5	
subitem not at all important	○	○	◉	○	○	essential

Selectie wissen

Does your paper address subitem 22-ii?

Copy and paste relevant sections from the manuscript (include quotes in quotation marks "like this" to indicate direct quotes from your manuscript), or elaborate on this item by providing additional information not in the ms, or briefly explain why the item is not applicable/relevant for your study

Jouw antwoord

20) Trial limitations, addressing sources of potential bias, imprecision, and, if relevant, multiplicity of analyses

20-i) Typical limitations in ehealth trials

Typical limitations in ehealth trials: Participants in ehealth trials are rarely blinded. Ehealth trials often look at a multiplicity of outcomes, increasing risk for a Type I error. Discuss biases due to non-use of the intervention/usability issues, biases through informed consent procedures, unexpected events.

	1	2	3	4	5	
subitem not at all important	○	○	◉	○	○	essential

Selectie wissen

Does your paper address subitem 20-i? *

Copy and paste relevant sections from the manuscript (include quotes in quotation marks "like this" to indicate direct quotes from your manuscript), or elaborate on this item by providing additional information not in the ms, or briefly explain why the item is not applicable/relevant for your study

Yes, "ossible explanations for the lack of short-term effectiveness, e.g., the relatively large proportion of patients with a low risk of developing persistent LBP included in the analysis who have a favorable natural prognosis and the fact that blended care is not suitable for all patients, also apply to the findings of this study and have been discussed in detail previously29." AND "First, the content of the app (i.e., self-management information, integrated fortnightly reminders, and the continuing availability of the app) may have been insufficient to further support patients' self-management behavior in the home setting. On the other hand, the results of our qualitative study did reveal that patients with chronic LBP (i.e., a duration of LBP of more than 12 weeks at the start of the study) did show adequate self-management behavior when experiencing a relapse in LBP. In case of a relapse, patients indicated that they first tried to gain control over their new episode of LBP before contacting a healthcare professional. However, patients did indicate that one of the biggest struggles was to maintain adequate health behavior in the pain-free periods between relapses in LBP72. Thus, this could mean that to facilitate long-term behavioral change in patients' management of LBP, more personalized self-management support during and after treatment is needed." AND "A second limitation is that stratified blended physiotherapy is still considered a “black box”. Although we provided a two-days training for physiotherapists on the integration of the app within face-to-face physiotherapy, we have no insight in the actual fidelity of the intervention, i.e., the degree to which the intervention is delivered as intended. Possibly, low fidelity has contributed to the absence of (cost-)effectiveness of e-Exercise LBP compared to face-to-face physiotherapy."

21) Generalisability (external validity, applicability) of the trial findings

NPT: External validity of the trial findings according to the intervention, comparators, patients, and care providers or centers involved in the trial

21-i) Generalizability to other populations

Generalizability to other populations: In particular, discuss generalizability to a general Internet population, outside of a RCT setting, and general patient population, including applicability of the study results for other organizations

	1	2	3	4	5	
subitem not at all important	○	○	◉	○	○	essential

Selectie wissen

Does your paper address subitem 21-i?

Copy and paste relevant sections from the manuscript (include quotes in quotation marks "like this" to indicate direct quotes from your manuscript), or elaborate on this item by providing additional information not in the ms, or briefly explain why the item is not applicable/relevant for your study

Jouw antwoord

21-ii) Discuss if there were elements in the RCT that would be different in a routine application setting

Discuss if there were elements in the RCT that would be different in a routine application setting (e.g., prompts/reminders, more human involvement, training sessions or other co-interventions) and what impact the omission of these elements could have on use, adoption, or outcomes if the intervention is applied outside of a RCT setting.

	1	2	3	4	5	
subitem not at all important	○	○	◉	○	○	essential

Selectie wissen

Does your paper address subitem 21-ii?

Copy and paste relevant sections from the manuscript (include quotes in quotation marks "like this" to indicate direct quotes from your manuscript), or elaborate on this item by providing additional information not in the ms, or briefly explain why the item is not applicable/relevant for your study

Jouw antwoord

OTHER INFORMATION

23) Registration number and name of trial registry

Does your paper address CONSORT subitem 23? *

Copy and paste relevant sections from the manuscript (include quotes in quotation marks "like this" to indicate direct quotes from your manuscript), or elaborate on this item by providing additional information not in the ms, or briefly explain why the item is not applicable/relevant for your study

Yes, "Trial Registration: ISRCTN 94074203; https://doi.org/10.1186/ISRCTN94074203"

24) Where the full trial protocol can be accessed, if available

Does your paper address CONSORT subitem 24? *

Cite a Multimedia Appendix, other reference, or copy and paste relevant sections from the manuscript (include quotes in quotation marks "like this" to indicate direct quotes from your manuscript), or elaborate on this item by providing additional information not in the ms, or briefly explain why the item is not applicable/relevant for your study

Yes, "Trial Registration: ISRCTN 94074203; https://doi.org/10.1186/ISRCTN94074203" and "Details on the design and methods of the trial were published previously28."

25) Sources of funding and other support (such as supply of drugs), role of funders

Does your paper address CONSORT subitem 25? *

Copy and paste relevant sections from the manuscript (include quotes in quotation marks "like this" to indicate direct quotes from your manuscript), or elaborate on this item by providing additional information not in the ms, or briefly explain why the item is not applicable/relevant for your study

Yes, "This study was co-funded by the Taskforce for Applied Research SIA (RAAK.PRO02.063), part of the Dutch Research Council (NWO). The funder had no role in the study design, data collection and analysis, decision to publish, or preparation of the manuscript."

X27) Conflicts of Interest (not a CONSORT item)

X27-i) State the relation of the study team towards the system being evaluated

In addition to the usual declaration of interests (financial or otherwise), also state the relation of the study team towards the system being evaluated, i.e., state if the authors/evaluators are distinct from or identical with the developers/sponsors of the intervention.

	1	2	3	4	5	
subitem not at all important	○	○	◉	○	○	essential

Selectie wissen

Does your paper address subitem X27-i?

Copy and paste relevant sections from the manuscript (include quotes in quotation marks "like this" to indicate direct quotes from your manuscript), or elaborate on this item by providing additional information not in the ms, or briefly explain why the item is not applicable/relevant for your study

Yes, no conflicts of interest were declared

About the CONSORT EHEALTH checklist

As a result of using this checklist, did you make changes in your manuscript? *

- ○ yes, major changes
- ○ yes, minor changes
- ◉ no

What were the most important changes you made as a result of using this checklist?

Jouw antwoord

How much time did you spend on going through the checklist INCLUDING making changes in your manuscript *

30 to 60 minutes in total

As a result of using this checklist, do you think your manuscript has improved? *

- ○ yes
- ◉ no
- ○ Anders:

Would you like to become involved in the CONSORT EHEALTH group?

This would involve for example becoming involved in participating in a workshop and writing an "Explanation and Elaboration" document

- ○ yes
- ◉ no
- ○ Anders:

Selectie wissen

Any other comments or questions on CONSORT EHEALTH

Jouw antwoord

STOP - Save this form as PDF before you click submit

To generate a record that you filled in this form, we recommend to generate a PDF of this page (on a Mac, simply select "print" and then select "print as PDF") before you submit it.

When you submit your (revised) paper to JMIR, please upload the PDF as supplementary file.

Don't worry if some text in the textboxes is cut off, as we still have the complete information in our database. Thank you!

Final step: Click submit !

Click submit so we have your answers in our database!

Verzenden

Formulier wissen

Verzend nooit wachtwoorden via Google Formulieren.

Deze content is niet gemaakt of goedgekeurd door Google. Misbruik rapporteren - Servicevoorwaarden - Privacybeleid

Google Formulieren
