# Supplementary figures and images for: Effectiveness and Cost-Effectiveness of a Stratified Blended Physiotherapy Intervention Compared With Face-to-Face Physiotherapy in Patients With Nonspecific Low Back Pain: Cluster Randomized Controlled Trial
Source: J Med Internet Res. 2023 Nov 24;25:e43034. doi: 10.2196/43034 (PMC10709796; doi:10.2196/43034)

## Multimedia Appendix 3. Print screens of the smartphone application

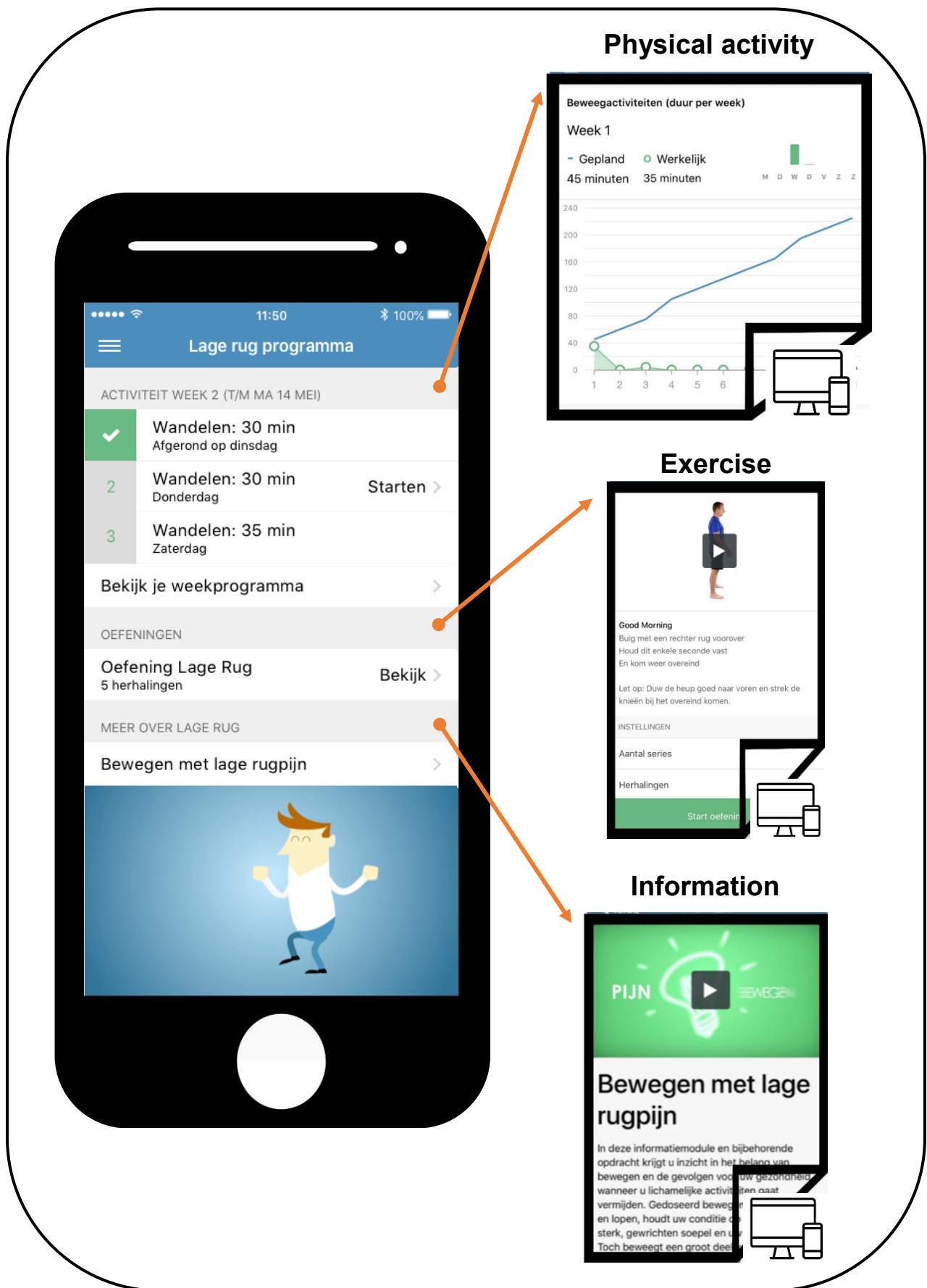

Supplement: Multimedia Appendix 3 [file jmir_v25i1e43034_app3.pdf]
