# Supplementary material for: Effectiveness and Cost-Effectiveness of a Stratified Blended Physiotherapy Intervention Compared With Face-to-Face Physiotherapy in Patients With Nonspecific Low Back Pain: Cluster Randomized Controlled Trial
Source: J Med Internet Res. 2023 Nov 24;25:e43034. doi: 10.2196/43034 (PMC10709796; doi:10.2196/43034)
Supplement: Multimedia Appendix 4 [file jmir_v25i1e43034_app4.docx]

## Multimedia Appendix 4. Within-group changes in the stratified blended physiotherapy group and the face-to-face physiotherapy group for the primary and secondary clinical outcome measures during the 12-month follow-up

|  | | Stratified blended physiotherapy | | | Face-to-face physiotherapy | | |
| --- | --- | --- | --- | --- | --- | --- | --- |
| Outcome | | (n=102) | | | (n=102) | | |
|  | | Mean | Within-group changes | | Mean | Within-group changes | |
|  | | 95% CI | 95% CI | *P* value | 95% CI | 95% CI | *P* value |
|  | |  |  |  |  |  |  |
|  | **Physical functioning (ODI, 0-100)** | | | | | |  |
|  | Baseline | 19.4 (16.3 to 22.4) | - | - | 20.2 (17.5 to 22.9) | - | - |
|  | 3 months | 9.7 (6.6 to 12.7) | -9.7 (-13.3 to -6.1) | <.001 | 9.4 (7.0 to 11.9) | -10.7 (-13.7 to -7.8) | <.001 |
|  | 12 months | 8.0 (5.3 to 10.6) | -11.4 (-14.9 to -8.0) | <.001 | 8.9 (6.0 to 11.7) | -11.3 (-14.7 to -8.0) | <.001 |
|  | **Utility score (EQ-5D-5L, 0-1)** | | | | | |  |
|  | Baseline | 0.729 (0.692 to 0.765) | - | - | 0.751 (0.725 to 0.776) | - | - |
|  | 3 months | 0.847 (0.813 to 0.880) | 0.118 (0.076 to 0.160) | <.001 | 0.841 (0.806 to 0.876) | 0.090 (0.055 to 0.126) | <.001 |
|  | 12 months | 0.851 (0.803 to 0.900) | 0.123 (0.067 to 0.178) | <.001 | 0.840 (0.791 to 0.889) | 0.089 (0.042 to 0.135) | <.001 |
|  | **Average pain intensity in past 7 days (NRS, 0-10)** | | | | | |  |
|  | Baseline | 5.7 (5.3 to 6.0) | - | - | 5.4 (5.0 to 5.8) | - | - |
|  | 3 months | 3.2 (2.7 to 3.8) | -2.4 (-3.0 to -1.9) | <.001 | 3.0 (2.5 to 3.4) | -2.5 (-3.0 to -1.9) | <.001 |
|  | 12 months | 2.4 (1.8 to 2.9) | -3.3 (-3.9 to -2.8) | <.001 | 2.7 (2.2 to 3.2) | -2.7 (-3.2 to -2.2) | <.001 |
|  | **Physical activity (Activ8, MVPA min/d)** | | | | | |  |
|  | Baseline | 80.1 (71.5 to 88.7) | - | - | 74.1 (65.6 to 82.7) | - | - |
|  | 3 months | 76.2 (67.0 to 85.3) | -4.0 (-13.1 to 5.2) | .39 | 69.7 (62.1 to 77.2) | -4.4 (-11.4 to 2.6) | .22 |
|  | 12 months | 76.7 (65.8 to 87.7) | -3.4 (-14.4 to 7.6) | .54 | 70.4 (62.2 to 78.7) | -3.7 (-11.7 to 4.4) | .37 |
|  | **Fear avoidance beliefs (FABQ, 0-96)** | | | | | |  |
|  | Baseline | 28.1 (24.9 to 31.2) | - | - | 25.4 (22.2 to 28.5) | - | - |
|  | 3 months | 23.3 (20.5 to 26.2) | -4.8 (-7.0 to -2.5) | <.001 | 25.0 (21.6 to 28.4) | -0.3 (-2.5 to 1.8) | .75 |
|  | 12 months | 21.5 (18.1 to 24.9) | -6.6 (-10.3 to -2.9) | <.001 | 24.0 (20.5 to 27.4) | -1.4 (-4.2 to 1.5) | .34 |
|  | **Pain catastrophizing (PCS, 0-52)** | | | | | |  |
|  | Baseline | 11.1 (9.2 to 13.0) | - | - | 10.3 (8.6 to 12.0) | - | - |
|  | 3 months | 9.1 (7.5 to 10.8) | -2.0 (-3.5 to -0.4) | .01 | 9.3 (7.3 to 11.3) | -1.0 (-2.5 to 0.5) | .19 |
|  | 12 months | 7.9 (6.2 to 9.6) | -3.2 (-5.1 to -1.3) | .00 | 8.2 (6.6 to 9.9) | -2.1 (-3.5 to -0.6) | .01 |
|  | **Self-efficacy (GSE Scale, 10-40)** | | | | | |  |
|  | Baseline | 32.0 (31.2 to 32.9) | - | - | 33.1 (32.4 to 33.8) | - | - |
|  | 3 months | 31.9 (31.0 to 32.8) | -0.1 (-0.8 to 0.6) | .77 | 32.6 (31.9 to 33.4) | -0.5 (-1.2 to 0.2) | .20 |
|  | 12 months | 32.6 (31.7 to 33.4) | 0.5 (-0.4 to 1.4) | .26 | 33.0 (32.2 to 33.9) | -0.1 (-0.9 to 0.7) | .84 |
|  | **Patient activation (PAM 13-Dutch, 0-100)** | | | | | |  |
|  | Baseline | 62.5 (60.0 to 64.9) | - | - | 64.7 (62.2 to 67.2) | - | - |
|  | 3 months | 61.9 (59.5 to 64.4) | -0.5 (-3.0 to 2.0) | .67 | 64.5 (61.9 to 67.1) | -0.3 (-2.8 to 2.3) | .84 |
|  | 12 months | 65.6 (62.5 to 68.6) | 3.1 (-0.4 to 6.6) | .08 | 64.0 (61.2 to 66.9) | -0.7 (-3.4 to 2.0) | .61 |
|  | **Patient self-reported adherence to prescribed home exercises (EARS, 0-24)** ^a^ | | | | | |  |
|  | Baseline | - | - | - | - | - | - |
|  | 3 months | 11.9 (11.4 to 12.5) | N/A | N/A | 11.1 (10.7 to 11.6) | N/A | N/A |
|  | 12 months | 12.4 (11.8 to 13.1) | N/A | N/A | 12.2 (11.7 to 12.7) | N/A | N/A |

^a^ Patient self-reported adherence to prescribed home exercises could only be measured after the treatment period

^b^ ODI = Oswestry Disability Index

^c^ NRS = Numeric Rating Scale

^d^ MVPA = Moderate to Vigorous Physical Activity

^e^ min/d = minutes per day

^f^ FABQ = Fear Avoidance Beliefs Questionnaire

^g^ PCS = Pain Catastrophizing Scale

^h^ GSE = General Self-efficacy Scale

^i^ PAM = Patient Activation Measure

^j^ EARS = Exercise Adherence Rating Scale

^k^ N/A = Not Applicable
