# Supplementary material for: Effectiveness and Cost-Effectiveness of a Stratified Blended Physiotherapy Intervention Compared With Face-to-Face Physiotherapy in Patients With Nonspecific Low Back Pain: Cluster Randomized Controlled Trial
Source: J Med Internet Res. 2023 Nov 24;25:e43034. doi: 10.2196/43034 (PMC10709796; doi:10.2196/43034)
Supplement: Multimedia Appendix 5 [file jmir_v25i1e43034_app5.docx]

## Multimedia Appendix 5. Mean costs per participant in the stratified blended physiotherapy group and the face-to-face physiotherapy group during the 12-month follow-up for complete cases and per risk group for developing persistent low back pain

|  | | Complete cases | |
| --- | --- | --- | --- |
| Cost category | | Cost per participant (€),  mean (SEM) ^c^ | |
|  | | Stratified blended physiotherapy | Face-to-face physiotherapy |
|  | | (n=82) | (n=89) |
|  |  |  |  |
| **Healthcare** ^a^ | | 370 (54) | 384 (40) |
|  | Intervention | 200 (12) | 211 (11) |
|  | Primary healthcare excluding intervention | 130 (26) | 117 (23) |
|  | Secondary healthcare | 36 (29) | 51 (18) |
|  | Medication | 5 (2) | 5 (2) |
| Informal care | | 218 (78) | 275 (79) |
| Absenteeism FCA ^d^ | | 504 (259) | 572 (242) |
| Absenteeism HCA ^e^ | | 542 (290) | 1455 (1106) |
| Presenteeism | | 1732 (359) | 2176 (462) |
| Unpaid Productivity | | 403 (97) | 300 (72) |
| **Societal FCA ^b^** | | 3228 (557) | 3708 (560) |
| **Societal HCA ^b^** | | 3267 (575) | 4590 (1201) |

|  | | Risk of developing persistent low back pain | | | | | |
| --- | --- | --- | --- | --- | --- | --- | --- |
| Cost category | | Low Risk | | Medium Risk | | High Risk | |
|  | | Cost per participant (€),  mean (SEM) ^c^ | | Cost per participant (€),  mean (SEM) ^c^ | | Cost per participant (€),  mean (SEM) ^c^ | |
|  | | Stratified blended physiotherapy | Face-to-face physiotherapy | Stratified blended physiotherapy | Face-to-face physiotherapy | Stratified blended physiotherapy | Face-to-face physiotherapy |
|  | | (n=58) | (n=62) | (n=34) | (n=37) | (n=10) | (n=3) |
|  |  |  |  |  |  |  |  |
| **Healthcare** ^a^ | | 385 (86) | 387 (50) | 653 (94) | 536 (96) | 766 (185) | 330 (84) |
|  | Intervention | 163 (17) | 205 (14) | 251 (21) | 248 (19) | 337 (42) | 260 (76) |
|  | Primary healthcare excluding intervention | 150 (42) | 118 (29) | 304 (74) | 205 (64) | 334 (132) | 48 (12) |
|  | Secondary healthcare | 66 (47) | 59 (28) | 85 (43) | 72 (33) | 80 (105) | 0 (0) |
|  | Medication | 7 (3) | 5 (2) | 13 (4) | 11 (4) | 15 (12) | 23 (16) |
| Informal care | | 322 (152) | 249 (92) | 987 (318) | 747 (247) | 1193 (610) | 1944 (1161) |
| Absenteeism FCA ^d^ | | 489 (418) | 348 (236) | 1206 (755) | 1197 (636) | 949 (756) | 0 (0) |
| Absenteeism HCA ^e^ | | 318 (239) | 277 (123) | 1012 (648) | 3241 (2653) | 702 (424) | 0 (0) |
| Presenteeism | | 2096 (729) | 2499 (738) | 3423 (1691) | 3473 (992) | 5402 (2798) | 2733 (2733) |
| Unpaid Productivity | | 369 (166) | 225 (102) | 1376 (426) | 712 (219) | 2400 (767) | 1090 (555) |
| **Societal FCA ^b^** | | 3661 (1102) | 3708 (925) | 7646 (2356) | 6665 (1228) | 10711 (3742) | 6097 (4170) |
| **Societal HCA ^b^** | | 3490 (964) | 3637 (867) | 7451 (2298) | 8709 (2779) | 10464 (3747) | 6097 (4170) |

^a^ Healthcare costs are the sum of the primary healthcare costs, secondary healthcare costs, medication costs and intervention costs

^b^ Societal costs are the sum of the healthcare costs, informal care costs, absenteeism costs, presenteeism costs and unpaid productivity costs

^c^ Costs are expressed in 2020 Euros

^d^ FCA = Friction cost approach

^e^ HCA = Human capital approach
